# Adverse life events and cognition in older adults: the role of gender and psychological resilience

**DOI:** 10.1093/geronb/gbag100

**Published:** 2026-06-08

**Authors:** Yvonne Leung, Ranmalee Eramudugolla, Moyra E Mortby, Kaarin J Anstey

**Affiliations:** School of Psychology, University of New South Wales, Sydney, New South Wales, Australia; School of Behavioural and Health Sciences, Faculty of Health Sciences, Australian Catholic University, Strathfield, New South Wales, Australia; School of Psychology, University of New South Wales, Sydney, New South Wales, Australia; School of Psychology, University of New South Wales, Sydney, New South Wales, Australia; Mortby Consulting, Canberra, Australian Capital Territory, Australia; School of Psychology, University of New South Wales, Sydney, New South Wales, Australia; Neuroscience Research Australia, Randwick, New South Wales, Australia; (Psychological Sciences Section)

**Keywords:** Traumatic experience, Life events, Cognitive impairment, Dementia, Cognitive aging

## Abstract

**Objectives:**

This study examined gender differences in the association between stressful life events and late-life cognition, with psychological resilience as a potential moderating factor.

**Methods:**

Longitudinal data were drawn from a population-based sample of 1,815 participants (*M*_age_ = 70.71, *SD*_age_ = 1.50, 48% women). Stressful life events were indicated by both the number of recent life events in the past 6 months measured by the List of Threatening Events Questionnaire and lifetime traumatic experiences captured by questions from the Composite International Diagnostic Interview. Cognitive outcomes included neuropsychological assessments of incident dementia and mild cognitive impairment (MCI) and domain-specific performance. Psychological resilience was measured by the Connor–Davidson Resilience Scale. Logistic and linear models were conducted, adjusting for common risk factors.

**Results:**

Lifetime trauma was associated with higher MCI (*OR* = 1.78, *p* < .001) and dementia risk (*OR* = 1.18, *p* = .02), and poorer performance in memory and executive function. Gender differences were found. Psychological resilience was more strongly associated with better executive function in men than in women (*B* = 0.03, *p* = .04, 95% CI [0.00, 0.07]). It also buffers the impact of lifetime trauma on working memory, and the effect was stronger in men than in women (*B* = 0.11, *p* = .04).

**Discussion:**

The impact of stressful life events on cognition may differ by gender, potentially reflecting gender-specific types of stressors and coping mechanisms. Further investigations on the social and individual factors that contribute to psychological resilience and their role in cognitive aging are warranted.

There is growing evidence that exposure to stressful experiences can lead to a cascade of psychological and physiological responses that negatively affect brain and cognitive function. However, relatively little is known about moderating factors such as gender and psychological resilience. Approximately 25% of healthy older adults experience at least one stressful life event within a three-month period (Ormel et al., 2001). These events have been linked to late-life cognitive decline (Comijs et al., 2011) and an increased risk of dementia (Franks et al., 2021). In particular, exposure to two or more stressful life events, compared to none, was significantly associated with a heightened risk of all-cause dementia (Franks et al., 2021).

Severs et al. (2023) recently published the first systematic and meta-analytic review on the association between traumatic life events and dementia risk. Of the studies included, only 5 considered events occurring during adulthood, with 3 focusing specifically on war-related experience. While these studies consistently suggest that traumatic life events elevate dementia risk, none have investigated the potential moderating roles of gender and psychological resilience. This article aimed to address this gap.

Psychological trauma was defined as an exposure to “actual or threatened death, serious injury, or sexual violence” (American Psychiatric Association, 2013). More broadly, it refers to highly stressful and impactful events that are often unexpected and uncontrollable. Such experiences may include natural disasters and wars, as well as situations that exceed one’s ability to cope cognitively and emotionally (Wang et al., 2023). These experiences can include discrete events (or psychological stressors) such as divorce (either as a child with parents who are divorcing or an adult who directly experienced it), bereavement, socioeconomic deprivation, vital exhaustion (Bougea et al., 2022), and other events in various forms of physical and emotional abuse or neglect.

Emerging studies have observed associations between adverse life events and late-life cognition. For instance, Zuelsdorff et al. (2024) found that increased exposure to adulthood trauma was strongly associated with poorer baseline cognition across Black and White participants. Among White participants, childhood traumatic experience was linked to poorer baseline cognition. Similarly, Lynch and Lachman (2020) reported that individuals with more lifetime trauma exposure had a greater decline in executive function and episodic memory over a 9-year period. Nilaweera et al. (2020) indicated that lifetime major trauma and re-experiencing post-traumatic stress disorder symptoms were associated with low global cognition, but this was observed in women only. Although the number of psychological stressors has been associated with increased risk of dementia and Alzheimer’s disease, few studies have explored gender differences in these associations, with most reporting no significant differences (Bougea et al., 2022). It is plausible, however, that gender differences exist in both the types of life events individuals are exposed to and in their psychological and physiological response to these events. Differences in cognitive outcomes by gender may be partially explained by these differences in the way individuals cope with and adapt to adversity, and how they were impacted by those stressful life events. Such variations may help explain observed gender disparities in cognitive decline in later life.

The mechanisms underlying the relationship between adverse life events and late-life cognition remain uncertain. One proposed pathway involves individual differences in perceived stress, which may influence brain function through the action of stress hormones such as glucocorticoids, and indirectly through behavioral coping strategies (Belanoff et al., 2001; Franks et al., 2021). Higher levels of perceived stress have been associated with increased risk of mild cognitive impairment (MCI) and all-cause dementia (Franks et al., 2021). Stress may also contribute to dementia risk indirectly due to its associations with poorer mental health and increased engagement in lifestyle risk factors such as smoking, alcohol use, and poor sleep quality (Seib et al., 2014). At the same time, there are documented sex differences in stress reactivity and responses (Verma et al., 2011). Stress reactivity is commonly indicated by activities of the Hypothalamic–Pituitary–Adrenal (HPA) axis and sympathetic nervous systems, such as heart rate and blood pressure, and there are sex-specific patterns of HPA and autonomic reactivity and regulation (Hodes et al., 2024). Women showed stronger HPA and inflammatory responses to stress with broader and more complex pathways, while men exhibit a tighter coupling of physiological and behavioral responses, and mechanisms that facilitate recovery. These differences may contribute to variations in the way traumatic experiences influence cognitive outcomes across genders and suggest factors such as psychological resilience which may operate differently in men and women.

Psychological resilience factors may moderate the relationship between stress and cognitive decline, as well as physiological markers of aging. Psychological resilience refers to the capacity to withstand or recover from exposure to adverse circumstances that pose significant threats to an individual’s stability, viability, or development (Masten, 2011; Rutter, 2006). It has been shown to buffer the negative impact of perceived stress on depressive symptoms (da Silva-Sauer et al., 2021) and to moderate the relationship between stress and biological aging. For instance, individuals with lower resilience exhibit greater stress-related physiological age acceleration, whereas higher resilience attenuates the association between stress and markers of aging such as insulin resistance (Harvanek et al., 2021). Resilient older adults showed lower levels of hopelessness and depression, higher functional independence, improved resistance to stress, and better cognition (MacLeod et al., 2016). A recent review by James et al. (2023) also highlighted the interactions between resilience, age, and sex in stress response. Advancing age was associated with higher resilience and lower stress response, while females exhibit a higher level of resilience, even though they tend to experience greater chronic stress and more minor daily stressors than males. Given sex and gender differences in stress reactivity, coping strategies and social roles, psychological resilience may differentially buffer the impact of trauma on late-life cognition in men and women.

Several studies have reported relationships between psychological resilience and better cognitive performance in older adults (e.g., Jung et al., 2021; McDaniel et al., 2022; Saez-Sanz et al., 2023). However, few have examined whether resilience moderates the association between stressful life events and cognition. Moreover, gender differences in psychological resilience and cognitive outcomes have been documented in various studies (e.g., Lou et al., 2023; Phillips et al., 2016), yet the interplay between gender, stressful life events, and cognition remains less well understood. In particular, it is unclear whether psychological resilience may moderate the effect of adverse life events on cognitive outcomes differently across genders.

In this article, we aimed to investigate gender differences in the association between adverse life events—both recent and lifetime—and cognition in older adults, with psychological resilience as a potential moderating factor. This investigation is important for several reasons. First, there is a notable lack of longitudinal research examining the relationship between adverse life events and cognitive outcomes in older adults; to date, only two studies have been identified, and one of them had a sample drawn from a clinical trial (Lynch & Lachman, 2020; Nilaweera et al., 2023). Second, no prior study has explored whether psychological resilience moderates the association between traumatic experience and cognition, despite evidence suggesting that individuals with higher psychological resilience tend to exhibit better cognitive function than their counterparts (Jung et al., 2021; McDaniel et al., 2022; Yang et al., 2021). Third, past studies have typically examined a narrow range of life events, with some focusing on war experience and others on domestic violence or spousal bereavement (Severs et al., 2023). Finally, gender differences may exist in the relationship between stressful life events and cognitive decline, as prior research indicates that the association between psychological resilience and cognitive impairment may be stronger among women (Lou et al., 2023).

We hypothesize that: (a) adverse life events would have a negative impact on cognition; (b) psychological resilience would moderate the relationship between life events and cognition, such that the impact of life events on cognitive outcomes would be weaker among individuals with higher resilience. (c) Gender would moderate the association between life events and cognition. (d) The moderating effect of resilience in the link between adverse life events and cognition would differ by gender.

## Method

### Participants

We report data of 1,815 participants (48% women) from the Personality and Total Health Through life Project (PATH). PATH is a longitudinal population study, and its design has been described elsewhere (Anstey et al., 2021; Anstey et al., 2012). Residents (aged 60–64 at recruitment) of the city of Canberra and the adjacent town of Queanbeyan in Australia were randomly sampled through the electoral roll (response rate = 58.3%) and reassessed every 4 years since 2001. This study analyzed data from wave 3 (2009–10) and 4 (2013–14) of PATH where data on psychological resilience, recent life events, and lifetime traumatic experience were available (Supplementary Figure 1). Here, we refer to wave 3 as “baseline” as we are using data from wave 3 to predict cognitive performance in wave 4. Participants were excluded if they met the criteria for MCI (*n* = 50) or dementia (*n* = 7) diagnosis at baseline (see Eramudugolla et al., 2017 for more details). According to the power analysis for a linear model with 13 predictor variables (including the covariates), power = 0.80, effect size = 0.20, at a significance level of .05, the estimated minimum sample size is 101. The sample size of the existing dataset exceeds the minimum suggested sample size by the power analysis. The study is approved by the Australian National University and the University of New South Wales Human Ethics Committee. The study was conducted in compliance with institutional research standards and with the Helsinki Declaration. All participants had provided written informed consent.

### Measures

#### Recent life events and lifetime traumatic experience

Life events experienced within the previous 6 months were measured at each wave using the List of Threatening Events Questionnaire (Brugha & Cragg, 1990) [test–retest reliability (*k* = 0.78–1.0), validity (*k* = 0.63–0.83)]. The current analysis only included recent life events assessed in wave 3. It consists of 16 items, and participants were asked if each of the listed life events or problems happened to them during the last 6 months. Examples include “Your parent, child, or partner died” and “You had a major financial crisis.” The total number of events was calculated. In total, 10 questions about lifetime traumatic experience were adopted from the Composite International Diagnostic Interview (World Health Organization, 1993) in which data collected at wave 3 were used. Some examples of questions include “Did you ever have direct combat experience in a war?” and “Have you ever been threatened with a weapon, held captive, or kidnapped?” Participants responded either “Yes” or “No” to each question, and the total number of experiences was calculated (see Supplementary Methods). While these items are derived from a well-established interview, we acknowledge that they do not constitute a formally validated, standalone scale.

#### Psychological resilience and gender

Psychological resilience was measured by the Connor-Davidson Resilience Scale (Cronbach’s α = 0.89) (Connor & Davidson, 2003) and was administered at wave 3. Participants were asked to indicate the degree to which they agree on each of the 25 items (on a 5-point range from “Not true at all” [score of 0] to “True nearly all the time” [score of 4]) as each applies to them over the last month. The total score ranges from 0 to 100, with higher scores implying greater psychological resilience. Gender was self-reported with the option of “male” and “female,” and this data is undifferentiated between biological sex and social gender (Bauer, 2023).

#### Cognition

Cognition was assessed using a neuropsychological battery. Global cognition was measured by the Mini-Mental State Exam (MMSE) (Folstein et al., 1975). Memory and Working Memory were measured by the California Verbal Learning Test-Immediate Recall (Anstey et al., 2012; Delis et al., 2000) and the Digit Span Backwards (Wechsler, 1945) respectively. Oral version of the Symbol Digit Modalities Test (SDMT) (Smith, 1973) was used to measure Processing Speed, while Executive Function was measured by the Trail Making Test (parts A and B) (Reitan & Wolfson, 1985). Please note that lower scores in the Trail Making Tests indicate better performance.

MCI and dementia diagnoses were determined based on performance on a neurocognitive test battery, informant reports, health and medical conditions, using Petersen et al. (Petersen et al., 1999), Winblad et al. (Winblad et al., 2004), and DSM-4 criteria. Participants who had poor cognitive performance at waves 1 to 3 were invited to complete a detailed neurocognitive assessment, an MRI scan, and a blood test for clinical diagnosis of dementia or MCI (Anstey et al., 2012). Further information can be found in Supplementary Methods and Eramudugolla et al. (2017).

#### Measurement of covariates

Baseline covariates related to cognition included in the models are age in years, total years of education (measured in wave 2 of PATH), household income, depression, apolipoprotein E (*APOE*) *e4* carrier status, hypertension, diabetes, smoking (currently smoke?), non-English speaking background, and physical exercise. Annual household income (in Australian dollars) was categorized and ordered (lowest-1, highest-6) into ≤$16,000, $16,001-$30,000, $30,001-$56,000, $56,001-$88,000, $88,001-$125,000, or >$125,000. Depression/depressive symptoms was assessed by the 9-item Goldberg Depression Scale (Goldberg et al., 1988), and it was treated as a continuous variable in the analysis. Physical activity was self-reported and categorized by vigour (mild, moderate, and vigorous) (Australian Institute of Health and Welfare, 2000). Hypertension and diabetes were self-reported, where participants were asked if they had been told by the doctor or a health professional that they have these conditions. *APOE* genotypes were determined using Taqman assays on genomic DNA obtained from buccal swabs, as previously described (Cherbuin et al., 2008).

### Statistical approach

Missing data were assessed across all study variables. Analyses included participants with complete data on psychological resilience, recent life events, and lifetime traumatic experience. Cases with missing data on variables included in a given model were excluded from that analysis. As a result, the sample size varied slightly across models.

Scores in each cognitive test, years of education, scores from the Goldberg Depression Scale, the total number of lifetime traumatic experiences, and those of recent life events were transformed into *z*-scores. Age at baseline was centered to the mean. Descriptive statistics were computed to represent sample characteristics. Gender group differences in recent life events and lifetime traumatic experience were examined using independent sample *t*-tests.

Logistic regressions were conducted to examine the association between recent life events/lifetime trauma at baseline and the risk of incident cognitive impairment at wave 4. Distinct models were performed to analyze incident MCI and incident dementia as separate outcomes. Linear regression models were used to examine the relationships between recent life events/lifetime trauma and each continuous cognitive outcome.

We first fitted main effects models to examine associations between recent life events/lifetime trauma and each outcome. Second, interaction terms between gender and recent life events/lifetime trauma were added to examine gender differences. Concurrently, interaction terms between resilience and recent life events/lifetime trauma were included in the models. Finally, three-way interaction terms between gender, recent life events/lifetime trauma, and resilience were added to explore if the moderating effect of resilience differs by gender.

Both unadjusted and adjusted models were estimated. Adjusted models included the following covariates: baseline cognition (of the domain examined in that model), baseline age, total years of education, hypertension, diabetes, depression, smoking status, apolipoprotein E (*APOE*) e4 carrier status, physical activity, and non-English speaking background. Generalised Variance Inflation Factor (GVIF) was calculated for each predictor variable to examine multicollinearity (Supplementary Table 1). Sensitivity analyses were conducted with separate models including the covariates only. Statistical analyses were conducted in RStudio (version 2023.06). This study and its analysis plan were preregistered (Leung et al., 2024).

## Results

### Sample characteristics

Supplementary Figure 1 presents the participant flow and how the final analytic sample varied based on the availability of data on recent life events, lifetime trauma, and resilience. In our sample, close to 7% of the participants were from a non-English speaking background (see Table 1). There were no gender differences in the number of recent life events (*t*(1804) = 1.70, *p *= .089, 95% CI [−0.01, 0.21]), but men had significantly more lifetime traumatic experiences than women (*t*(1813) = −10.83, *p* < .001, 95% CI [−0.76, −0.53]) (Table 1). Women experienced certain traumatic experiences at a younger age than men, such as combat experience (note: the number for women in this category is small compared to men), while more men experienced serious physical assault at a younger age than women (Supplementary Table 2). There were significant gender differences in the frequency of each type of lifetime traumatic experience. For instance, more men reported experiencing military combat, life-threatening accidents, natural disasters, witnessing bad injury or death, or serious physical assault. More women reported experiencing rape or sexual molestation (Table 2). For recent life events that happened in the past six months, more women than men reported experiencing the death of a partner, parent, or child. More women than men also reported serious problems with a friend in the past six months (Table 3). Women had significantly higher scores in psychological resilience than men; *t*(1687) = 2.26, *p* = .024, 95% CI [0.19, 2.77].

**Table 1:** Baseline characteristics (wave 3 sample) with changes in cognitive performance from wave 3 to 4.

Characteristics	Male (*n *= 943)				Female (*n *= 872)				
	Mean	*SD*	*n*	%	Mean	*SD*	*n*	%	
**Baseline (Wave 3)**									
** Age**	70.55	1.47			70.67	1.52			
** Education**	14.52	2.71			13.61	2.58			
** Household income**									
1-lowest			33	3.62			91	11.73	
2			181	19.87			245	31.57	
3			325	35.68			254	32.73	
4			230	25.25			128	16.49	
5			84	9.22			43	5.54	
6-highest			58	6.37			15	1.93	
** Non-English-speaking background**			76	8.14			45	5.25	
** Hypertension**			500	53.71			433	50.29	
** Diabetes**			147	15.62			76	8.73	
** Currently smoke**			48	5.10			50	5.73	
** Physical activity**									
None/mild			353	37.67			435	50.46	
Moderate			404	43.12			343	39.79	
Vigorous			180	19.21			84	9.74	
** Goldberg’s Depression Scale (range: 0–9)**	1.50	1.77			1.71	1.81			
** Life-time traumatic experience (range: 0–10)**	1.56	1.40			0.91	1.10			
** Recent life events (range: 0–16)**	0.74	1.15			0.84	1.23			
** Psychological resilience (range: 0–100)**	72.81	13.05			74.29	13.89			
** MMSE (range: 0–30)**	29.07	1.27			29.29	1.02			
** Immediate recall (range: 0–16)**	6.23	2.00			7.27	2.31			
** Digit span backward**	5.27	2.19			4.99	2.21			
** Trail making test A (time in seconds)**	36.97	14.87			35.70	11.45			
** Trail Making Test B (time in seconds)**	82.56	33.99			85.60	32.92			
** Symbol digit modalities test (range: 0–110)**	47.74	9.07			48.48	9.02			
**Follow-up (Wave 4)**									
MCI at wave 4	65	8.38			53	7.73			
Dementia at wave 4	11	1.42			10	1.40			
**Cognitive score/time change from baseline to follow-up**									
MMSE	−2.17	14.72			−3.48	18.22			
Immediate recall	−0.43	1.47			−0.24	1.39			
Digit span backwards	−1.23	1.98			−1.68	2.21			
Trail making test A (time in seconds)	0.04	2.04			0.10	2.09			
Trail making test B (time in seconds)	−2.48	6.00			−2.44	6.76			
Symbol digit modalities test	12.89	38.10			10.25	36.46			

MMSE, mini-mental state examination; MCI, mild cognitive impairment. Sample sizes vary across analyses due to missing data and the availability of wave 4 outcomes. Cognitive change scores represent the differences between wave 3 and wave 4. Positive values indicate improvement for MMSE, immediate recall, digit span backward, and symbol digit modalities test, whereas for trail making test A and B, negative values reflect improvement.

**Table 2:** Frequency of lifetime traumatic experience by gender.

Life-time Traumatic Experience	*N*	Male		Female		Total		*X* ^2^	*p*
		*n*	%	*n*	%	*n*	%		
**Combat experience**	1,812	82	8.7	8	0.9	90	5.0	58.24	<.001
**Life threatening accident**	1,812	276	29.3	124	14.2	400	22.1	59.91	<.001
**Natural disaster**	1,811	279	29.6	180	20.7	459	25.3	19.18	<.001
**Witnessed bad injury or death**	1,812	429	45.6	168	19.3	597	32.9	141.64	<.001
**Ever raped**	1,806	6	0.6	34	3.9	40	2.2	22.43	<.001
**Ever sexually molested**	1,806	94	10.0	132	15.2	226	12.5	11.20	<.001
**Seriously physically assaulted**	1,811	101	10.7	45	5.2	146	8.1	18.86	<.001
**Threatened with weapon, held captive, kidnapped**	1,812	120	12.8	36	4.1	156	8.6	42.71	<.001
**Tortured or victim of terrorists**	1,812	9	1.0	2	0.2	11	0.6	3.96	.05
**Other**	1,808	437	46.5	402	46.3	839	46.4	0.01	.94

**Table 3:** Frequency of recent life events in the past six months by gender.

Life events	*N*	Male		Female		Total		*X* ^2^	*p*
		*n*	%	*n*	%	*n*	%		
**You yourself suffered a serious illness**	1,813	93	9.9	92	10.6	185	10.2	0.24	.63
**A serious illness+ happened to a close relative**	1,813	135	14.3	148	17.0	283	15.6	2.43	.12
**Your parent, child, or partner died**	1,813	35	3.7	50	5.7	85	4.7	4.15	.04
**A close family friend or another relative died**	1,813	217	23.0	200	23.0	417	23.0	0.00	.97
**You broke off a steady relationship**	1,812	8	0.8	8	0.9	16	0.9	0.03	.87
**You had serious problem with a friend**	1,812	56	5.9	87	10.0	143	7.9	10.23	.00
**You had a crisis or disappointment in your work**	1,813	17	1.8	13	1.5	30	1.7	0.27	.60
**You thought you would soon lose your job**	1,809	17	1.8	13	1.5	30	1.7	0.28	.60
**Your partner thought he/she would soon lose job**	1,367	11	1.3	7	1.3	18	1.3	0.01	.92
**Your partner had a crisis or disappointment in his/her work**	1,367	18	2.2	17	3.1	35	2.6	1.10	.30
**You had a separation due to marital difficulties**	1,368	3	0.4	4	0.7	7	0.5	0.86	.35
**You became unemployed or you were seeking work unsuccessfully**	1,811	10	1.1	5	0.6	15	0.8	1.32	.25
**You were sacked from your job**	1,811	4	0.4	4	0.5	8	0.4	0.01	.91
**You had a major financial crisis**	1,811	43	4.6	38	4.4	81	4.5	0.04	.84
**You had problems with the police and a court appearance**	1,813	5	0.5	9	1.0	14	0.8	1.49	.22
**Something you valued was lost or stolen**	1,813	30	3.2	37	4.2	67	3.7	1.44	.23

Main effects models without interaction terms are presented in Supplementary Table 3, representing associations between recent life events/lifetime trauma, gender, psychological resilience, and cognitive outcomes after adjusting for covariates. As analyses were performed using complete-case data, they resulted in smaller sample sizes compared to the baseline sample presented in Table 1. Across these models, lifetime trauma was associated with poorer cognitive outcomes. Recent life events, gender, and psychological resilience did not show consistent main effects. Fully adjusted models with interaction terms are presented in Supplementary Table 4. Main effects in these models represent conditional effects and should be interpreted accordingly. Results presented below are based on fully adjusted models, with main effects of recent life events and lifetime trauma interpreted based on models without interaction terms (see Table 4).

**Table 4:** Summaries of models examining the association between life events/traumatic experience and cognition after adjusting for covariates.

Cognitive Tests	Recent life events					Lifetime traumatic experience				
	Variables	*B*	*SE*	*p*	95% CI	Variables	*B*	*SE*	*p*	95% CI
**MMSE**	Life events	−0.01	0.04	.83	−0.08, 0.06	Traumatic experience	−0.04	0.04	.37	−0.13, 0.05
	**Gender (ref: Female)**	−0.27	0.05	**<.001**	−0.36, −0.17	**Gender**	−0.25	0.05	**<.001**	−0.35, −0.15
	Psychological resilience	0.02	0.03	.58	−0.05, 0.08	Psychological resilience	0.01	0.04	.83	−0.06, 0.08
	Gender × Life events	−0.06	0.05	.24	−0.15, 0.04	Gender × Traumatic experience	0.04	0.05	.49	−0.07, 0.14
	Psychological resilience × Life events	−0.00	0.03	.51	−0.06, 0.06	Psychological resilience × Traumatic experience	−0.04	0.05	.41	−0.13, 0.05
	Gender × Psychological resilience	−0.03	0.05	.51	−0.12, 0.06	Gender × Psychological resilience	−0.03	0.05	.60	−0.13, 0.07
	Gender × Life events × Psychological resilience	0.04	0.05	.48	−0.06, 0.14	Gender × Traumatic experience × Psychological resilience	0.02	0.05	.78	−0.09, 0.12
**Immediate Recall**	Life events	−0.02	0.04	.57	−0.10, 0.06	Traumatic experience	0.01	0.05	.88	−0.08, 0.10
	**Gender**	−0.15	0.05	**.01**	−0.26, −0.05	**Gender**	−0.15	0.06	**.01**	−0.26, −0.04
	Psychological resilience	0.00	0.04	.94	−0.07, 0.07	Psychological resilience	0.01	0.04	.88	−0.07, 0.08
	Gender × Life events	0.04	0.05	.48	−0.07, 0.14	Gender × Traumatic experience	0.04	0.06	.45	−0.15, 0.07
	Psychological resilience × Life events	0.03	0.04	.33	−0.03, 0.10	Psychological resilience × Traumatic experience	0.01	0.05	.81	−0.08, 0.10
	Gender × Psychological resilience	0.05	0.05	.31	−0.05, 0.15	Gender × Psychological resilience	0.06	0.05	.23	−0.04, 0.17
	Gender × Life events × Psychological resilience	−0.08	0.05	.12	−0.19, 0.02	Gender × Traumatic experience × Psychological resilience	−0.03	0.06	.57	−0.14, 0.08
**Digit Span Backwards**	Life events	0.03	0.04	.42	−0.05, 0.11	**Traumatic experience**	−0.11	0.05	**.02**	−0.20, −0.02
	Gender	0.05	0.05	.30	−0.05, 0.15	Gender	0.07	0.05	.19	−0.04, 0.17
	Psychological resilience	0.02	0.04	.64	−0.05, 0.09	Psychological resilience	0.01	0.04	.72	−0.06, 0.09
	Gender × Life events	−0.03	0.05	.61	−0.13, 0.08	Gender × Traumatic experience	0.10	0.05	.06	−0.00, 0.21
	Psychological resilience × Life events	0.02	0.03	.56	−0.05, 0.09	Psychological resilience × Traumatic experience	–0.04	0.05	.40	−0.13, 0.05
	**Gender × Psychological resilience**	−0.14	0.05	**.01**	−0.24, −0.04	**Gender × Psychological resilience**	–0.15	0.05	**.01**	−0.25, −0.05
	Gender × Life events × Psychological resilience	−0.02	0.05	.66	−0.13, 0.08	**Gender × Traumatic experience × Psychological resilience**	0.11	0.06	**.04**	0.01, 0.22
**Symbol Digit Modalities Test**	Life events	0.02	0.03	.49	−0.04, 0.08	Traumatic experience	−0.03	0.04	.36	−0.11, 0.04
	Gender	−0.05	0.04	.23	−0.13, 0.03	Gender	−0.03	0.04	.49	−0.11, 0.05
	Psychological resilience	0.03	0.03	.32	−0.03, 0.08	Psychological resilience	0.03	0.03	.33	–0.03, 0.09
	Gender × Life events	−0.00	0.04	.97	−0.08, 0.08	Gender × Traumatic experience	–0.01	0.04	.88	–0.09, 0.08
	Psychological resilience × Life events	0.01	0.03	.69	−0.04, 0.06	Psychological resilience × Traumatic experience	−0.01	0.04	.81	–0.08, 0.06
	Gender × Psychological resilience	−0.06	0.04	.16	−0.13, 0.02	Gender × Psychological resilience	–0.04	0.04	.29	–0.13, 0.04
	Gender × Life events × Psychological resilience	0.03	0.04	.56	−0.06, 0.11	Gender × Traumatic experience × Psychological resilience	–0.02	0.04	.65	–0.11, 0.07
**Trail Making Test A**	Life events	−0.01	0.01	.30	−0.04, 0.01	**Traumatic experience**	0.04	0.01	**.01**	0.01, 0.07
	Gender	0.02	0.02	.30	−0.02, 0.05	Gender	0.00	0.02	.87	–0.03, 0.04
	**Psychological resilience**	−0.02	0.01	**.03**	−0.05, −0.00	**Psychological resilience**	–0.03	0.01	**.02**	–0.05, –0.01
	Gender × Life events	0.02	0.02	.19	−0.01, 0.05	Gender × Traumatic experience	–0.03	0.02	.09	–0.06, 0.00
	Psychological resilience × Life events	0.01	0.01	.30	−0.01, 0.03	Psychological resilience × Traumatic experience	−0.01	0.01	.52	–0.04, 0.02
	**Gender × Psychological resilience**	0.03	0.02	**.05**	0.00, 0.06	**Gender × Psychological resilience**	0.03	0.02	**.04**	0.00, 0.07
	Gender × Life events × Psychological resilience	−0.02	0.02	.29	−0.05, 0.02	Gender × Traumatic experience × Psychological resilience	0.01	0.02	.45	–0.02, 0.05
**Trail making test B**	Life events	−0.01	0.01	.48	−0.04, 0.02	Traumatic experience	0.02	0.02	.32	–0.02, 0.05
	Gender	0.03	0.02	.10	−0.01, 0.07	Gender	0.02	0.02	.20	–0.01, 0.06
	Psychological resilience	−0.00	0.01	.75	−0.03, 0.02	Psychological resilience	0.00	0.01	.95	–0.03, 0.03
	Gender × Life events	0.02	0.02	.23	−0.01, 0.06	Gender × Traumatic experience	−0.00	0.02	.86	–0.04, 0.04
	Psychological resilience × Life events	0.02	0.01	.13	−0.01, 0.04	Psychological resilience × Traumatic experience	0.01	0.02	.41	–0.02, 0.05
	Gender × Psychological resilience	0.01	0.02	.62	−0.03, 0.04	Gender × Psychological resilience	0.00	0.02	1.00	–0.04, 0.04
	Gender × Life events × Psychological resilience	−0.01	0.02	.76	−0.05, 0.03	Gender × Traumatic experience × Psychological resilience	−0.00	0.02	.92	−0.04, 0.04

CI = confidence interval; MMSE = Mini-Mental State Examination; *SE* = standard error. Covariates: baseline age, total years of education, hypertension, diabetes, depression, smoking status, apolipoprotein E (*APOE) e4* carrier status, physical activity, and non-English speaking background. Significant associations (*p* < .05) are in bold.

### MCI And dementia risks

Logistic regression models were conducted to examine the associations between adverse life events and incident MCI and dementia. In the main effects model (Supplementary Table 3), recent life event, gender, and psychological resilience were not associated with MCI incidence. In the fully adjusted models including interaction terms (Supplementary Table 4), no significant interactions were observed. Several covariates were associated with higher risk of MCI, including lower education, depression, *APOE4* carriers (one allele vs none), and diabetes (with the largest effect size). On the other hand, lifetime trauma was associated with higher MCI risk (*OR *= 1.78, *p* <.001, β = 0.67, 95% CI [1.45, 2.17]), while gender and psychological resilience were not associated with MCI incidence. No interaction between lifetime trauma, gender, and resilience was observed. Lower education, *APOE4 (carrying one allele vs none)*, and diabetes were associated with higher risk of MCI. The fully adjusted models were not significant. Multicollinearity tests suggest that the correlations between predictor variables were weak, with GVIFs of each variable close to 1 (see Supplementary Table 1) (Fox & Monette, 1992).

Similar results of MCI risk, recent life event, gender, and psychological resilience were not associated with dementia incidence. While no interactions were found, lower education and carrying two alleles of *APOE4* were significantly associated with higher risk of dementia, with *APOE4* having the largest effect size. Lifetime trauma was associated with higher risk of dementia (*OR *= 1.18, *p *= .02, β = 0.60, 95% CI [1.10, 2.98]). Similar to recent life event, carrying two *APOE4* alleles predicts higher dementia risk. The fully adjusted models for both recent life event and lifetime trauma were not significant. In these models, estimates for some variables were unstable due to a low number of cases and should be interpreted with caution.

### Cognitive tests

Linear regression models examined the association between recent life events, lifetime trauma, gender, and psychological resilience with cognitive performance across multiple domains: MMSE, Immediate Recall, Digit Span Backward, Symbol Digit Modality Test, and Trail Making Test A and B, adjusting for covariates. Main effects models are presented in Supplementary Table 3, and interaction models in Supplementary Table 4. All the models presented below are statistically significant.

#### Mini-mental state exam

Both recent life event and lifetime trauma were not associated with MMSE. Compared to women, men showed worse MMSE score at follow-up (recent life event: *B *= −0.26, *p* < .001, β = −0.32, 95% CI [−0.36, −0.17]; lifetime trauma: *B *= −0.25, *p* < .001, β = −0.31, 95% CI [−0.35, −0.16]). No significant interactions were observed. Covariates, including baseline MMSE, education, and hypertension, significantly predict MMSE performance, with baseline performance having the largest effect size.

#### Immediate recall

Similar to the MMSE findings, both recent life event and lifetime trauma were not associated with Immediate Recall performance at follow-up. Men performed worse than women (Recent life event: *B *= −0.16, *p* = .004, β = −0.16, 95% CI [−0.26, −0.05]; Lifetime trauma: *B *= −0.15, *p *= .007, β = −0.15, 95% CI [−0.26, −0.04]). Age, baseline immediate recall performance, education, and physical exercises were associated with memory performance, where baseline performance showed the largest relative contribution to the prediction.

##### Digit span backward

**Figure 1:**
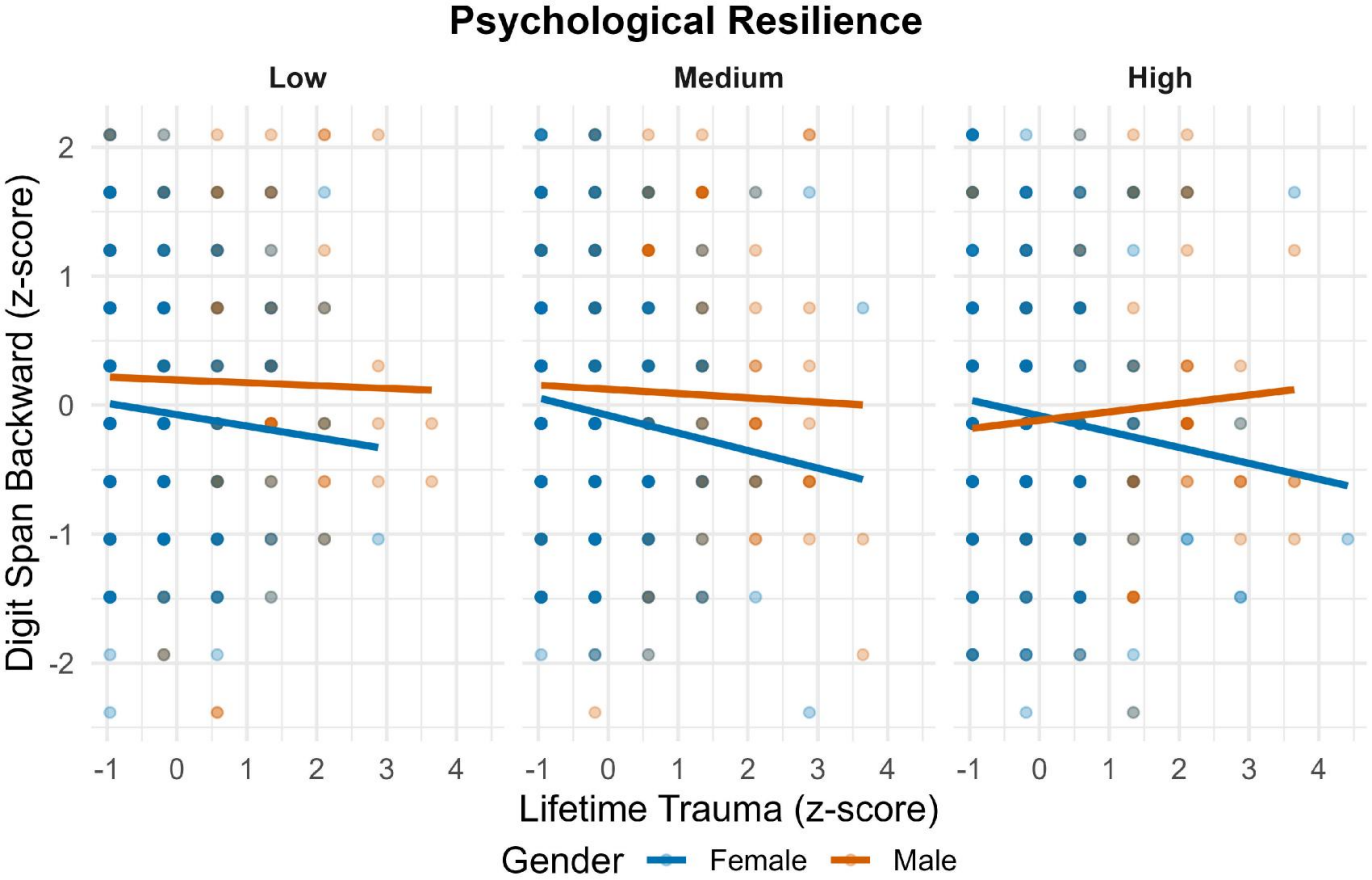
Associations between lifetime trauma and digit span backward scores (working memory), stratified by resilience level and gender. Lines represent linear trends. Psychological resilience was categorised into low, medium, and high groups based on tertiles of the standardized resilience scores.

Model suggested no association between recent life event and digit span backward score, but psychological resilience predicted worse performance (*B *= −0.05, *p *= .04, β = −0.05, 95% CI [−0.10, −0.00]). An interaction between gender and psychological resilience was observed (*B *= −0.14, *p *< .01, β = −0.13, 95% CI [−0.24, −0.04]), indicating that the positive relationship between psychological resilience and digit span backward score was weaker in men than in women. Lifetime trauma was associated with worse performance (*B *= −0.11, *p *= .02, β = −0.11, 95% CI [−0.20, −0.02]). The interaction between gender and psychological resilience was significant (*B *= −0.15, *p* < .01, β = −0.15, 95% CI [−0.25, −0.05]), indicating a weaker association between resilience and working memory in men than in women. Furthermore, a significant three-way interaction between gender, psychological resilience, and lifetime trauma was found (Figure 1) (*B *= 0.11, *p *= .04, *β* = 0.11, 95% CI [0.01, 0.22]), suggesting that higher psychological resilience attenuated the negative association between lifetime trauma and working memory, with this buffering effect appearing stronger in men than in women. As covariates, age, baseline performance, depression, and non-English speaking background were associated with score at follow-up, with baseline performance having the largest effect size.

#### Symbol digit modalities test

Main effects models suggest no significant association between recent life event or lifetime trauma and SDMT performance. Fully adjusted models suggest that baseline performance significantly predicts performance.

#### Trail making test A and B

While recent life event was not a significant predictor, lifetime traumatic experience was associated with worse Trail Making Test A performance (*B *= 0.02, *p *= .02, β = 0.06, 95% CI [0.00, 0.04]). A significant interaction between gender and psychological resilience was found in the recent life event model (*B *= 0.03, *p *= .049, β = 0.10, 95% CI [0.00, 0.06]), and in the lifetime traumatic event model (*B *= 0.03, *p *= .04, β = 0.11, 95% CI [0.00, 0.07]), indicating the association between resilience and cognitive score as stronger in men than in women. Baseline performance, depression, household income, and non-English speaking background were associated with test performance, with baseline test score having the largest effect size. In contrast, recent life event and lifetime traumatic experience were not associated with trail making test B performance. Fully adjusted models revealed significant covariates, including baseline performance, education, depression, and non-English speaking background, where baseline performance has the strongest predictive power.

## Discussion

This study examined the moderating role of psychological resilience in the relationship between stressful life events—both recent and over the lifetime—and cognition in older adults, with attention to gender differences. To our knowledge, this is among the first studies to investigate the role of psychological resilience in the relationship between adverse life events and late-life cognition using longitudinal data.

In our sample, men reported more lifetime traumatic experiences than women, while there was no significant difference in the number of recent adverse life events between genders. Certain lifetime traumatic experiences are more prevalent in men, such as military combat or serious physical assault, while others are more common in women, such as rape or sexual molestation. Women also had higher psychological resilience scores than men. Our results indicated that lifetime traumatic experience was associated with higher MCI risk and dementia, while no associations were found between recent life events and cognitive outcomes. Lifetime trauma was also associated with poorer performance in working memory and executive function tasks (motor speed and visual attention). We observed some evidence of interaction effects between gender and psychological resilience, as well as a three-way interaction between gender, lifetime trauma, and psychological resilience for working memory performance. However, we should interpret these interaction effects with caution given the number of tests conducted and the modest effect sizes. As psychological resilience increases, the impact of lifetime trauma on working memory becomes weaker, and this is more strongly observed in men compared to women. Contrary to our hypothesis, psychological resilience did not moderate the effect of lifetime trauma or recent life event on other cognitive outcomes. However, psychological resilience was associated with better executive functioning, and the correlation was stronger in men compared to women.

The differential impact of lifetime traumatic experiences and recent life events on late-life cognition may reflect discrepancies in the timing and accumulation of stress exposure. Lifetime trauma may capture cumulative stress across the lifespan, which may exert long-term effects on neurobiological systems involved in cognition. For instance, early-life adversity has been linked to prolonged glucocorticoid exposure, which may affect cognitive function in later life (McEwen, 2008b). In addition, early traumatic experiences may shape educational attainment, socioeconomic trajectories, and health behaviors, all of which are known risk factors for cognitive impairment (Richards & Wadsworth, 2004). In contrast, recent life events may exert more transient effects that are less likely to translate into measurable cognitive impairment. However, it is possible that recent life events may still exacerbate cognitive decline through cumulative stress effects, particularly among individuals with prior trauma (Comijs et al., 2011; McEwen, 2008a). Individuals with more lifetime traumatic experience may also respond to recent life events differently, potentially reflecting better adaptation or coping shaped by accumulated resilience. Consistent with this speculation, we observed associations between lifetime trauma and cognitive outcomes, whereas recent life events were not associated with cognitive performance, aligning with some prior research (Comijs et al., 2011; Franks et al., 2021; Severs et al., 2023). Nonetheless, findings in the literature are mixed. For example, Korten et al. (2014) found associations of both recent and childhood stressful events and slower processing speed, particularly among individuals with depressive symptoms. Rosnick et al. (2007) reported that only specific recent events (not the aggregate), such as injury or illness of a friend, and financial difficulties, were linked to cognitive performance. Further research is needed to clarify the mechanisms underlying these differences.

Gender differences were observed in the association between psychological resilience and cognitive outcomes, and the way psychological resilience moderated the impact of lifetime trauma. Notably, the interaction between gender and psychological resilience varies between cognitive tasks. For working memory, the association between resilience and performance was weaker in men, while in the test of executive function (motor speed and attention), the association was stronger in men compared to women. Psychological resilience was only found to moderate the association between lifetime trauma and working memory, and it was more profound in men than in women.

These findings provide tentative evidence regarding potential sex differences in the association between resilience, early life trauma, and cognition, although the observed patterns varied across cognitive domains and should be interpreted cautiously given the number of statistical tests conducted. One possible explanation is the differences in the types of traumatic experience by men and women, and the ways in which they respond to those stressors. For example, men in our sample reported higher exposure to trauma such as physical assault and combat, while women had higher exposure to interpersonal trauma. These differences might result in varied coping strategies and physiological responses across gender, thereby shaping how resilience operates in relation to late-life cognition. In past studies, such as Samplin et al. (2013), childhood abuse was associated with reduced hippocampus volume in males but not in females. Munro et al. (2019) found no gender differences in the relationship between traumatic experiences and cognition. Among older adults with lifetime trauma and posttraumatic stress disorder (PTSD) symptoms, women showed a 46% increased risk of low global cognition and poorer performance in verbal fluency and psychomotor speed (Nilaweera et al., 2020). Not all studies found a sex difference in cognitive performance in relation to lifetime trauma, and psychological resilience may help explain the previously mixed findings. Our current observations suggest that the buffering effect of psychological resilience may vary by gender, timing of the adverse event, and the cognitive domain we are examining. In particular, resilience may reduce the influence of lifetime trauma on working memory, particularly in men, although further research is needed to confirm these effects.

The literature on psychological resilience and cognitive aging has been inconclusive. Some studies have found psychological resilience to be associated with better cognitive outcomes, particularly in men with lower levels of education and older age (Yang et al., 2021), while others have reported it being associated with a lower risk of cognitive impairment in women (Jung et al., 2021). A recent review by Norbury et al. (2023) highlighted evidence where resilience buffers the impact of trauma on cognition through adaptive brain responses, in which individuals with higher resilience present greater activation in brain regions linked to cognitive control and emotion regulation. These neural patterns are associated with better late-life cognition, particularly in domains of executive function, working memory, and attention (e.g., van der Werff et al., 2013). However, most neuroimaging studies on resilience and trauma have not examined sex or gender differences. In our sample, women scored higher on psychological resilience than men, yet gender differences were only observed in executive function and working memory, moderated by psychological resilience. While not fully evaluated, the absence of a moderating effect of resilience on other cognitive domains or cognitive impairment might be due to the survivor effect, whereby individuals who have experienced traumatic events and remain physically and cognitively healthy may be inherently more resilient and possess greater socioeconomic and social resources. Psychological resilience may accumulate over time and is strengthened through personal experience and social support, which our current study was unable to fully capture. Taken together, the current findings highlight the importance of considering the role of gender and resilience when examining the influence of adverse life event on cognitive aging.

This study benefits from a large, population-based sample drawn from an Australian cohort, with comprehensive data on their socioeconomic, demographic, health, and cognitive status. A neuropsychological battery was administered, and dementia and MCI diagnoses were classified. However, the response rate (58%) may limit the generalizability of the findings. The study is also limited by the availability of data on psychological resilience at only one wave, preventing us from examining its development and impact on cognition over time. As resilience can evolve, a single time point might not adequately reflect an individual’s capacity to cope with lifetime adversity. Ongoing data collection in the PATH study will allow further exploration of these associations in future waves. Furthermore, cognitive reserve is known to protect against cognitive impairment and dementia. A majority of our participants were highly educated and may have performed better in the cognitive tests than the general population who have had similar traumatic experiences. Although the current sample size exceeded the minimum required for the main effects analysis, this study may have been underpowered to detect interaction effects. Therefore, findings for interaction terms should be interpreted cautiously. We also acknowledge the limitation that while our sample included participants from a non-English speaking background, all measurements were administered in English, and that we have not assessed the cross-cultural/language validation formally.

## Conclusion

This article revealed the complex interplay between psychological resilience, gender, and lifetime trauma on late-life cognition. Lifetime trauma showed a larger impact on cognition than recent adverse life events. Our findings suggest that psychological resilience may buffer the influence of lifetime trauma on cognition, particularly in men, although further research is needed to confirm these effects. This contributes to and supports the existing neuroimaging evidence on neural responses to trauma and their link to cognition. Further investigation to explore gender-related differences in the psychosocial factors for resilience and their implications for cognitive aging is warranted.

## Supplementary Material

gbag100_Supplementary_Data

## Data Availability

The data that support the findings of this study are not publicly available due to privacy reasons but are available from the data sharing committee upon reasonable request (more information can be found on http://www.pathstudy.org.au/). This study is preregistered on the Open Science Framework (Leung et al., 2024).
