# Supplementary material for: Adverse life events and cognition in older adults: the role of gender and psychological resilience
Source: J Gerontol B Psychol Sci Soc Sci. 2026 Jun 8;81(8):gbag100. doi: 10.1093/geronb/gbag100 (PMC13372685; doi:10.1093/geronb/gbag100)
Supplement: gbag100_Supplementary_Data [file gbag100_supplementary_data.docx]

**The Journals of Gerontology, Series B: Psychological Sciences and Social Sciences Supplementary Material: Leung, Eramudugolla, Mortby, & Kaarin J Anstey. Adverse life events and cognition in older adults: The role of gender and psychological resilience.**

**Supplementary Figure 1. Participant sampling.**


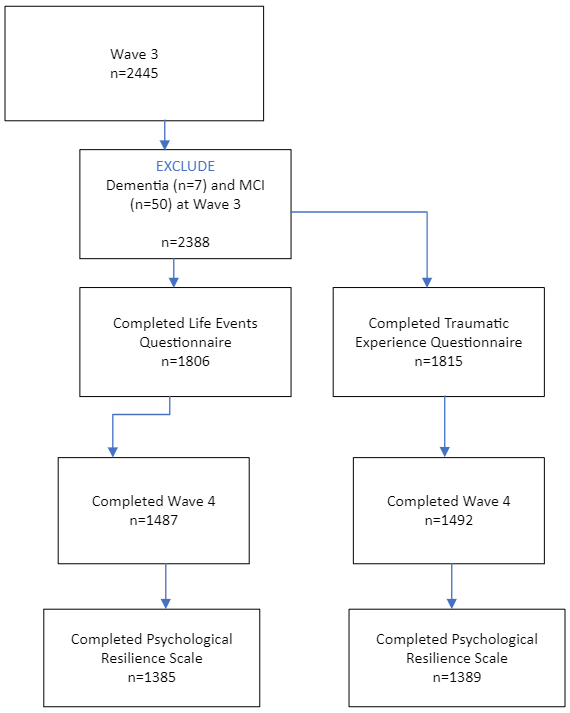


Note. Psychological resilience was measured at Wave 3.

**Supplementary Methods**

**Assessments**

**Questions on recent life events**

*Have any of the following life events or problems happened to you during the last six months?*

Response options: Yes/No

1. You yourself suffered a serious illness, injury or an assault.
2. A serious illness, injury or assault happened to a close relative.
3. Your parent, child or partner died.
4. A close family friend or another relative (aunt, cousin, grandparent) died.
5. You broke off a steady relationship.
6. You had a serious problem with a close friend, neighbour or relative.
7. You had a crisis or serious disappointment in your work or career.
8. You thought you would soon lose your job.
9. Your partner thought he/she would soon lose their job.
10. Your partner had a crisis or serious disappointment in his/her work or career.
11. You had a separation due to marital difficulties.
12. You became unemployed or you were seeking work unsuccessfully for more than one month.
13. You were sacked from your job.
14. You had a major financial crisis.
15. You had problems with the police and a court appearance.
16. Something you valued was lost or stolen.

* Skip items 9 to 10 if you are not currently married or living with a partner. However, do complete the following questions if your current spouse / partner is living in a nursing home or hostel.

**Questions on lifetime traumatic experience**

Response options: Yes/No; How old were you when you were last in this situation?

1. Did you ever have direct combat experience in a war?
2. Were you ever involved in a life-threatening accident?
3. Were you ever involved in a fire, flood or other natural disaster?
4. Did you ever witness someone badly injured or killed?
5. Were you ever raped? (that is, someone had sexual intercourse with you when you did not want to, by threatening you, or using some degree of force?)
6. Were you ever sexually molested (that is, someone touched or felt your genitals when you did not want them to)?
7. Were you ever seriously physically attacked or assaulted?
8. Have you ever been threatened with a weapon, held captive, or kidnapped?
9. Have you ever been tortured or the victim of terrorists?
10. Have you ever experienced any other extremely stressful or upsetting event?

**Mild Cognitive Impairment (MCI) and Dementia Diagnosis**

At waves 1-3, MCI was determined using a two-stage process (see Sargent-Cox et al., 2011) in which participants were screened for further assessment if they had any of the following: “(1) an MMSE score<25; (2) a score below the 5th percentile on immediate or delayed recall of the first trial of the California Verbal Learning Test (immediate or delayed score of <4 and <2, respectively), or (3) a score below the 5th percentile on either of the following two tests: Symbol-Digit Modalities Test ( <33) or Purdue Pegboard with both hands (wave 1: <8; wave 2 <7) or reaction time (third set of 20 trials; wave 1: >310 ms; wave 2: > 378 ms).” (Sargent-Cox et al., 2011, pp. 46-47). In the second phase, participants were selected based on the screening completed an in-person structured clinical assessment for dementia by one of two physicians, a neuropsychological assessment, and the Clinical Dementia Rating (CDR) Scale (Morris, 1993). MCI was diagnosed using Petersen et al. (1999) and Winblad et al. (2004) criteria. In wave 4, a modified 2-stage process was used (see Eramudugolla et al., 2017) in which all participants completed an extended in-person neuropsychological battery as well as informant ratings of cognition, function, and medical history. All data were then used in a psychometric algorithm to flag potential diagnoses, and a case file was created for each participant. A physician then reviewed flagged case files and classified participants where they met the criteria for MCI (Winblad et al., 2004).

DSM-IV criteria were used to assess dementia (Eramudugolla et al., 2017). In waves 1-3, clinical assessment involving a structured clinical assessment for dementia was performed by one of two physicians, together with a neuropsychological assessment and the CDR Scale (Morris, 1993). In wave 4, clinicians reviewed case files reporting on the cognitive data, medical background, and data provided by informants/family. Consensus diagnoses were formulated based on clinical checklists, data from neuropsychological assessments, and the participants' neuropsychiatric and medical histories (Anstey et al., 2008).

**Supplementary Table 1. Generalized Variance Inflation Factor (GVIF) of each predictor variable in the models.**

a.

|  | **GVIF** | **Df** |
| --- | --- | --- |
| Life Events | 1.03 | 1 |
| Gender | 1.14 | 1 |
| Resilience | 1.07 | 1 |
| MMSE | 1.10 | 1 |
| Age | 1.03 | 1 |
| Education | 1.28 | 1 |
| Depression | 1.15 | 1 |
| APOE e4 | 1.02 | 2 |
| Household Income | 1.23 | 1 |
| Hypertension | 1.04 | 1 |
| Diabetes | 1.07 | 1 |
| Currently smoke | 1.02 | 1 |
| Non-English Speaking Background | 1.03 | 1 |
| Physical Exercise | 1.12 | 2 |

b.

|  | **GVIF** | **Df** |
| --- | --- | --- |
| Lifetime Traumatic Experience | 1.11 | 1 |
| Gender | 1.21 | 1 |
| Resilience | 1.08 | 1 |
| Age | 1.03 | 1 |
| MMSE | 1.09 | 1 |
| Education | 1.27 | 1 |
| Depression | 1.15 | 1 |
| APOE e4 | 1.03 | 2 |
| Household Income | 1.22 | 1 |
| Hypertension | 1.04 | 1 |
| Diabetes | 1.08 | 1 |
| Currently smoke | 1.02 | 1 |
| Non-English Speaking Background | 1.03 | 1 |
| Physical Activity | 1.12 | 2 |

**Supplementary Table 2. Life-time Traumatic Experiences – the age that the participants were in the situation.**

1. All participants

|  | **n** | **Mean** | **SD** | **Min.** | **Max.** |
| --- | --- | --- | --- | --- | --- |
| Combat Experience | 89 | 26.47 | 9.40 | 2 | 47 |
| Life threatening accident | 395 | 36.41 | 17.37 | 3 | 74 |
| Natural disaster | 455 | 44.10 | 20.59 | 1 | 73 |
| Witnessed bad injury or death | 586 | 36.20 | 17.39 | 4 | 73 |
| Ever raped | 38 | 21.89 | 11.13 | 8 | 53 |
| Ever sexually molested | 220 | 14.00 | 8.83 | 4 | 60 |
| Seriously physically assaulted | 144 | 34.47 | 16.47 | 7 | 72 |
| Threatened with weapon, held captive, kidnapped | 153 | 36.20 | 16.59 | 5 | 71 |
| Tortured or victim of terrorists | 11 | 36.36 | 21.00 | 7 | 65 |
| Other | 829 | 50.65 | 18.27 | 1 | 73 |

1. By gender

|  | **Male** | | | | | **Female** | | | | | **Male vs Female** | |
| --- | --- | --- | --- | --- | --- | --- | --- | --- | --- | --- | --- | --- |
|  | **n** | **Mean** | **SD** | **Min.** | **Max.** | **n** | **Mean** | **SD** | **Min.** | **Max.** | ***F*** | ***p*** |
| Combat Experience | 81 | 27.88 | 7.54 | 6 | 44 | 8 | 12.25 | 14.41 | 2 | 47 | 25.79 | <.001 |
| Life threatening accident | 274 | 35.59 | 16.97 | 3 | 72 | 121 | 38.26 | 18.18 | 4 | 74 | 1.99 | 0.16 |
| Natural disaster | 278 | 44.11 | 19.23 | 2 | 73 | 177 | 44.07 | 22.63 | 1 | 73 | 0.00 | 0.99 |
| Witnessed bad injury or death | 423 | 35.81 | 16.85 | 4 | 72 | 163 | 37.21 | 18.73 | 5 | 73 | 0.77 | 0.38 |
| Ever raped | 6 | 16.67 | 8.85 | 11 | 34 | 32 | 22.88 | 11.36 | 8 | 53 | 1.60 | 0.21 |
| Ever sexually molested | 94 | 13.11 | 5.97 | 4 | 55 | 126 | 14.67 | 10.43 | 4 | 60 | 1.71 | 0.19 |
| Seriously physically assaulted | 101 | 32.62 | 16.19 | 7 | 72 | 43 | 38.79 | 16.48 | 7 | 71 | 4.33 | 0.04 |
| Threatened with weapon, held captive, kidnapped | 119 | 36.56 | 16.09 | 6 | 70 | 34 | 34.91 | 18.43 | 5 | 71 | 0.26 | 0.61 |
| Tortured or victim of terrorists | 9 | 38.11 | 22.84 | 7 | 65 | 2 | 28.50 | 9.19 | 22 | 35 | 0.32 | 0.59 |
| Other | 434 | 50.11 | 17.69 | 4 | 73 | 395 | 51.25 | 18.88 | 1 | 73 | 0.81 | 0.37 |

**Model summaries**

**Supplementary Table 3. Summary of adjusted main effects models without interaction terms.**

Recent life events and MCI incidence (*n =* 1195)

| **Predictor** | **OR [95% CI]** | **p** | **Std. Beta** |
| --- | --- | --- | --- |
| (Intercept) | 0.06 [0.03, 0.13] | <.001 | -2.848 |
| Recent Life Events | 0.98 [0.78, 1.24] | 0.884 | -0.017 |
| Gender (ref: Female) | 1.02 [0.65, 1.62] | 0.918 | 0.024 |
| Psychological Resilience | 1.09 [0.87, 1.36] | 0.480 | 0.081 |
| Age | 1.08 [0.93, 1.25] | 0.299 | 0.116 |
| Education | 0.71 [0.56, 0.89] | 0.004 | -0.338 |
| Depression | 1.35 [1.09, 1.68] | 0.007 | 0.284 |
| APOE (4+/4- vs non-carrier) | 1.91 [1.20, 3.03] | 0.006 | 0.648 |
| APOE (4+/4+ vs non-carrier) | 2.73 [0.59, 12.68] | 0.200 | 1.005 |
| Household Income | 1.01 [0.83, 1.23] | 0.931 | 0.011 |
| Hypertension | 0.84 [0.54, 1.31] | 0.441 | -0.176 |
| Diabetes | 2.41 [1.39, 4.17] | 0.002 | 0.88 |
| Smoking | 1.44 [0.56, 3.66] | 0.450 | 0.361 |
| Non-English Speaking Background | 1.70 [0.69, 4.20] | 0.247 | 0.532 |
| Exercise (none/mild vs moderate) | 0.73 [0.44, 1.20] | 0.218 | -0.313 |
| Exercise (vigorous vs moderate) | 1.42 [0.76, 2.66] | 0.271 | 0.352 |

Multiple R-squared: 0.0003898; Adjusted R-squared: -0.01233; F-statistic: 0.03065 on 15 and 1179 DF; p-value: 1

Lifetime Trauma and MCI incidence (*n =* 1197)

| **Predictor** | **OR [95% CI]** | **p** | **Std. Beta** |
| --- | --- | --- | --- |
| (Intercept) | 0.06 [0.03, 0.13] | <.001 | -2.81 |
| **Lifetime Trauma** | 1.78 [1.45, 2.17] | <.001 | 0.573 |
| Gender (ref: Female) | 0.71 [0.43, 1.15] | 0.165 | -0.371 |
| Psychological Resilience | 0.99 [0.79, 1.25] | 0.940 | -0.005 |
| Age | 1.08 [0.93, 1.25] | 0.311 | 0.117 |
| **Education** | 0.70 [0.55, 0.88] | 0.003 | -0.365 |
| Depression | 1.22 [0.97, 1.53] | 0.089 | 0.187 |
| **APOE (4+/4- vs non-carrier)** | 1.79 [1.11, 2.87] | 0.016 | 0.59 |
| APOE (4+/4+ vs non-carrier) | 2.58 [0.52, 12.75] | 0.244 | 0.965 |
| Household Income | 1.01 [0.82, 1.23] | 0.954 | 0.145 |
| Hypertension | 0.89 [0.56, 1.41] | 0.612 | -0.12 |
| **Diabetes** | 2.29 [1.29, 4.04] | 0.004 | 0.816 |
| Smoking | 1.35 [0.51, 3.55] | 0.546 | 0.283 |
| Non-English Speaking Background | 1.72 [0.68, 4.40] | 0.254 | 0.553 |
| Exercise (none/mild vs moderate) | 0.78 [0.46, 1.30] | 0.332 | -0.252 |
| Exercise (vigorous vs moderate) | 1.43 [0.75, 2.71] | 0.275 | 0.35 |

Multiple R-squared: 0.0009904; Adjusted R-squared: -0.0117; F-statistic: 0.07806 on 15 and 1181 DF; p-value: 1

Recent life events and dementia incidence (*n =* 1195)

| **Predictor** | **OR [95% CI]** | **p** | **Std. Beta** |
| --- | --- | --- | --- |
| (Intercept) | 0.00 [0.00, 0.05] | <.001 | -6.172 |
| Recent Life Events | 0.51 [0.20, 1.29] | 0.156 | -0.667 |
| Gender (ref: Female) | 0.98 [0.29, 3.39] | 0.979 | -0.029 |
| Psychological Resilience | 0.75 [0.41, 1.35] | 0.337 | -0.325 |
| Age | 1.35 [0.90, 2.01] | 0.146 | 0.456 |
| Education | 1.82 [0.89, 3.73] | 0.101 | 0.611 |
| **Depression** | 1.70 [1.00, 2.89] | 0.049 | 0.482 |
| APOE (4+/4- vs non-carrier) | 3.29 [0.96, 11.27] | 0.057 | 1.176 |
| **APOE (4+/4+ vs non-carrier)** | 23.63 [3.67, 152.18] | <.001 | 3.09 |
| Household Income | 0.77 [0.44, 1.35] | 0.365 | -1.068 |
| Hypertension | 1.14 [0.34, 3.83] | 0.837 | 0.148 |
| Diabetes | 1.99 [0.46, 8.59] | 0.354 | 0.735 |
| Smoking | 0.00 [0.00, Inf] | 0.994 | -16.203 |
| Non-English Speaking Background | 0.00 [0.00, Inf] | 0.994 | -15.966 |
| Exercise (none/mild vs moderate) | 2.13 [0.56, 8.13] | 0.271 | 0.78 |
| Exercise (vigorous vs moderate) | 0.89 [0.09, 8.58] | 0.923 | 0.002 |

Multiple R-squared: 0.000104; Adjusted R-squared: -0.01262; F-statistic: 0.008178 on 15 and 1179 DF; p-value: 1

Lifetime trauma and dementia incidence (*n =* 1197)

| **Predictor** | **OR [95% CI]** | **p** | **Std. Beta** |
| --- | --- | --- | --- |
| (Intercept) | 0.01 [0.00, 0.05] | <.001 | -6.042 |
| **Lifetime Trauma** | 1.81 [1.10, 2.98] | 0.019 | 0.6 |
| Gender (ref: Female) | 0.71 [0.20, 2.59] | 0.606 | -0.342 |
| Psychological Resilience | 0.71 [0.39, 1.28] | 0.251 | -0.379 |
| Age | 1.35 [0.91, 2.01] | 0.130 | 0.466 |
| Education | 1.87 [0.93, 3.78] | 0.081 | 0.638 |
| Depression | 1.36 [0.79, 2.35] | 0.263 | 0.276 |
| APOE (4+/4- vs non-carrier) | 3.15 [0.91, 10.83] | 0.069 | 1.133 |
| **APOE (4+/4+ vs non-carrier)** | 26.15 [4.14, 165.31] | <.001 | 3.197 |
| Household Income | 0.79 [0.44, 1.40] | 0.417 | -1.09 |
| Hypertension | 1.18 [0.34, 4.05] | 0.795 | 0.164 |
| Diabetes | 1.68 [0.37, 7.74] | 0.504 | 0.553 |
| Smoking | 0.00 [0.00, Inf] | 0.994 | -15.802 |
| Non-English Speaking Background | 0.00 [0.00, Inf] | 0.994 | -16.019 |
| Exercise (none/mild vs moderate) | 2.29 [0.59, 8.90] | 0.230 | 0.887 |
| Exercise (vigorous vs moderate) | 0.81 [0.08, 7.88] | 0.856 | -0.175 |

Multiple R-squared: 0.0001986; Adjusted R-squared: -0.0125; F-statistic: 0.01564 on 15 and 1181 DF; p-value: 1

Recent life events and MMSE (*n =* 1105)

| **Predictor** | **Beta** | **95% CI** | **p** | **Std. Beta** |
| --- | --- | --- | --- | --- |
| (Intercept) | 0.11 | [-0.042, 0.263] | 0.156 | 0.133 |
| Recent Life Events | -0.037 | [-0.083, 0.009] | 0.111 | -0.045 |
| **Gender (ref: Female)** | -0.263 | [-0.356, -0.170] | <.001 | -0.324 |
| Psychological Resilience | 0 | [-0.046, 0.046] | 0.997 | 0 |
| MMSE at baseline | 0.286 | [0.230, 0.342] | <.001 | 0.291 |
| Age | -0.019 | [-0.049, 0.010] | 0.199 | -0.036 |
| **Education** | 0.136 | [0.085, 0.187] | <.001 | 0.165 |
| Depression | -0.048 | [-0.098, 0.002] | 0.060 | -0.056 |
| APOE (4+/4- vs non-carrier) | 0.031 | [-0.070, 0.133] | 0.546 | 0.039 |
| APOE (4+/4+ vs non-carrier) | -0.045 | [-0.451, 0.362] | 0.829 | -0.055 |
| Household Income | 0.013 | [-0.027, 0.052] | 0.532 | 0.028 |
| **Hypertension** | 0.099 | [0.010, 0.188] | 0.030 | 0.123 |
| Diabetes | -0.064 | [-0.203, 0.075] | 0.369 | -0.079 |
| Smoking | -0.008 | [-0.224, 0.208] | 0.943 | -0.015 |
| Non-English Speaking Background | -0.195 | [-0.404, 0.013] | 0.066 | -0.245 |
| Exercise (none/mild vs moderate) | -0.032 | [-0.130, 0.065] | 0.516 | -0.041 |
| Exercise (vigorous vs moderate) | 0.012 | [-0.119, 0.142] | 0.861 | 0.013 |

Multiple R-squared: 0.1789; Adjusted R-squared: 0.1668; F-statistic: 14.82 on 16 and 1088 DF; p-value: < 2.2e-16

Lifetime trauma and MMSE (*n =* 1107)

| **Predictor** | **Beta** | **95% CI** | **p** | **Std. Beta** |
| --- | --- | --- | --- | --- |
| (Intercept) | 0.115 | [-0.038, 0.269] | 0.141 | 0.138 |
| Lifetime Trauma | -0.017 | [-0.064, 0.030] | 0.471 | -0.021 |
| **Gender (ref: Female)** | -0.254 | [-0.351, -0.157] | <.001 | -0.311 |
| Psychological Resilience | -0.002 | [-0.048, 0.045] | 0.941 | -0.002 |
| Age | -0.02 | [-0.050, 0.010] | 0.19 | -0.037 |
| c_mmse_z | 0.291 | [0.236, 0.347] | <.001 | 0.294 |
| Education | 0.13 | [0.079, 0.181] | <.001 | 0.156 |
| Depression | -0.048 | [-0.099, 0.002] | 0.061 | -0.055 |
| APOE (4+/4- vs non-carrier) | 0.036 | [-0.066, 0.139] | 0.486 | 0.045 |
| APOE (4+/4+ vs non-carrier) | -0.051 | [-0.461, 0.359] | 0.807 | -0.061 |
| Household Income | 0.012 | [-0.028, 0.052] | 0.564 | 0.031 |
| Hypertension | 0.091 | [0.001, 0.180] | 0.047 | 0.112 |
| Diabetes | -0.061 | [-0.201, 0.080] | 0.396 | -0.075 |
| Smoking | -0.013 | [-0.230, 0.204] | 0.907 | -0.021 |
| Non-English Speaking Background | -0.258 | [-0.466, -0.050] | 0.015 | -0.318 |
| Exercise (none/mild vs moderate) | -0.04 | [-0.138, 0.058] | 0.428 | -0.05 |
| Exercise (vigorous vs moderate) | 0.01 | [-0.121, 0.142] | 0.878 | 0.011 |

Multiple R-squared: 0.1805; Adjusted R-squared: 0.1685; F-statistic: 15 on 16 and 1090 DF; p-value: < 2.2e-16

Recent life events and immediate recall (*n =* 1183)

| **Predictor** | **Beta** | **95% CI** | **p** | **Std. Beta** |
| --- | --- | --- | --- | --- |
| (Intercept) | 0.004 | [-0.167, 0.175] | 0.965 | 0.138 |
| Recent Life Events | -0.009 | [-0.060, 0.041] | 0.714 | -0.009 |
| **Gender (ref: Female)** | -0.157 | [-0.264, -0.050] | 0.004 | -0.155 |
| Psychological Resilience | 0.033 | [-0.018, 0.083] | 0.210 | 0.033 |
| **Immediate recall at baseline** | -0.057 | [-0.090, -0.024] | <.001 | -0.087 |
| **Age** | 0.405 | [0.351, 0.458] | <.001 | 0.406 |
| **Education** | 0.153 | [0.098, 0.208] | <.001 | 0.157 |
| Depression | -0.011 | [-0.066, 0.045] | 0.707 | -0.01 |
| APOE (4+/4- vs non-carrier) | -0.106 | [-0.219, 0.007] | 0.067 | -0.105 |
| APOE (4+/4+ vs non-carrier) | -0.207 | [-0.630, 0.215] | 0.336 | -0.208 |
| Household Income | 0.037 | [-0.008, 0.081] | 0.109 | 0.067 |
| Hypertension | 0.024 | [-0.075, 0.123] | 0.635 | 0.025 |
| Diabetes | -0.057 | [-0.212, 0.098] | 0.471 | -0.059 |
| Smoking | -0.071 | [-0.317, 0.175] | 0.573 | -0.084 |
| Non-English Speaking Background | -0.168 | [-0.402, 0.067] | 0.161 | -0.175 |
| Exercise (none/mild vs moderate) | 0.004 | [-0.105, 0.113] | 0.938 | 0.001 |
| **Exercise (vigorous vs moderate)** | -0.175 | [-0.320, -0.029] | 0.019 | -0.182 |

Multiple R-squared: 0.2594; Adjusted R-squared: 0.2493; F-statistic: 25.53 on 16 and 1166 DF; p-value: < 2.2e-16

Lifetime trauma and immediate recall (*n =* 1185)

| **Predictor** | **Beta** | **95% CI** | **p** | **Std. Beta** |
| --- | --- | --- | --- | --- |
| (Intercept) | 0.003 | [-0.168, 0.175] | 0.968 | 0.131 |
| Lifetime Trauma | -0.021 | [-0.072, 0.031] | 0.434 | -0.021 |
| **Gender (ref: Female)** | -0.151 | [-0.261, -0.041] | 0.007 | -0.148 |
| Psychological Resilience | 0.033 | [-0.018, 0.084] | 0.207 | 0.034 |
| **Age** | -0.057 | [-0.090, -0.024] | <.001 | -0.088 |
| **Immediate recall at baseline** | 0.407 | [0.354, 0.460] | <.001 | 0.406 |
| **Education** | 0.155 | [0.100, 0.211] | <.001 | 0.158 |
| Depression | -0.007 | [-0.062, 0.048] | 0.808 | -0.007 |
| APOE (4+/4- vs non-carrier) | -0.094 | [-0.207, 0.020] | 0.106 | -0.093 |
| APOE (4+/4+ vs non-carrier) | -0.208 | [-0.632, 0.216] | 0.336 | -0.208 |
| Household Income | 0.035 | [-0.010, 0.080] | 0.124 | 0.066 |
| Hypertension | 0.025 | [-0.074, 0.125] | 0.618 | 0.026 |
| Diabetes | -0.058 | [-0.213, 0.098] | 0.465 | -0.06 |
| Smoking | -0.076 | [-0.322, 0.171] | 0.547 | -0.089 |
| Non-English Speaking Background | -0.185 | [-0.418, 0.048] | 0.120 | -0.191 |
| Exercise (none/mild vs moderate) | 0.006 | [-0.104, 0.115] | 0.917 | 0.003 |
| **Exercise (vigorous vs moderate)** | -0.173 | [-0.319, -0.027] | 0.020 | -0.18 |

Multiple R-squared: 0.2613; Adjusted R-squared: 0.2512; F-statistic: 25.82 on 16 and 1168 DF; p-value: < 2.2e-16

Recent life events and digit span backward (*n =* 1151)

| **Predictor** | **Beta** | **95% CI** | **p** | **Std. Beta** |
| --- | --- | --- | --- | --- |
| (Intercept) | 0.008 | [-0.158, 0.174] | 0.927 | 0.012 |
| Recent Life Events | 0.011 | [-0.038, 0.060] | 0.663 | 0.011 |
| Gender (ref: Female) | 0.048 | [-0.051, 0.148] | 0.34 | 0.046 |
| **Psychological Resilience** | -0.053 | [-0.103, -0.003] | 0.039 | -0.052 |
| Digit span backward at baseline | 0.545 | [0.496, 0.594] | <.001 | 0.551 |
| Age | -0.035 | [-0.067, -0.003] | 0.03 | -0.052 |
| Education | 0.021 | [-0.033, 0.075] | 0.453 | 0.017 |
| Depression | -0.065 | [-0.119, -0.011] | 0.019 | -0.061 |
| APOE (4+/4- vs non-carrier) | -0.028 | [-0.138, 0.082] | 0.612 | -0.029 |
| APOE (4+/4+ vs non-carrier) | 0.24 | [-0.165, 0.644] | 0.245 | 0.24 |
| Household Income | -0.01 | [-0.053, 0.034] | 0.658 | -0.008 |
| Hypertension | 0.001 | [-0.095, 0.097] | 0.985 | 0.001 |
| Diabetes | -0.067 | [-0.216, 0.082] | 0.377 | -0.068 |
| Smoking | -0.049 | [-0.282, 0.184] | 0.677 | -0.047 |
| Non-English Speaking Background | -0.333 | [-0.560, -0.105] | 0.004 | -0.33 |
| Exercise (none/mild vs moderate) | 0.003 | [-0.102, 0.109] | 0.955 | 0.004 |
| Exercise (vigorous vs moderate) | -0.057 | [-0.198, 0.084] | 0.428 | -0.057 |

Multiple R-squared: 0.347; Adjusted R-squared: 0.3378; F-statistic: 37.66 on 16 and 1134 DF; p-value: < 2.2e-16

Lifetime trauma and digit span backward (*n =* 1153)

| **Predictor** | **Beta** | **95% CI** | **p** | **Std. Beta** |
| --- | --- | --- | --- | --- |
| (Intercept) | 0.003 | [-0.163, 0.169] | 0.971 | 0.003 |
| Lifetime Trauma | -0.041 | [-0.090, 0.009] | 0.108 | -0.04 |
| Gender (ref: Female) | 0.066 | [-0.036, 0.169] | 0.206 | 0.063 |
| Psychological Resilience | -0.047 | [-0.098, 0.003] | 0.064 | -0.046 |
| **Age** | -0.035 | [-0.067, -0.003] | 0.031 | -0.052 |
| **Digit span backward at baseline** | 0.544 | [0.495, 0.593] | <.001 | 0.55 |
| Education | 0.023 | [-0.031, 0.077] | 0.412 | 0.019 |
| Depression | -0.057 | [-0.111, -0.003] | 0.040 | -0.053 |
| APOE (4+/4- vs non-carrier) | -0.021 | [-0.131, 0.089] | 0.710 | -0.021 |
| APOE (4+/4+ vs non-carrier) | 0.242 | [-0.163, 0.646] | 0.241 | 0.242 |
| Household Income | -0.011 | [-0.054, 0.033] | 0.626 | -0.009 |
| Hypertension | 0 | [-0.096, 0.096] | 0.999 | 0 |
| Diabetes | -0.062 | [-0.211, 0.086] | 0.412 | -0.063 |
| Smoking | -0.046 | [-0.278, 0.187] | 0.701 | -0.043 |
| **Non-English Speaking Background** | -0.345 | [-0.570, -0.120] | 0.003 | -0.343 |
| Exercise (none/mild vs moderate) | -0.004 | [-0.109, 0.102] | 0.947 | -0.002 |
| Exercise (vigorous vs moderate) | -0.055 | [-0.196, 0.086] | 0.444 | -0.054 |

Multiple R-squared: 0.3488; Adjusted R-squared: 0.3397; F-statistic: 38.03 on 16 and 1136 DF; p-value: < 2.2e-16

Recent life event and symbol digit modalities test (*n =* 1104)

| **Predictor** | **Beta** | **95% CI** | **p** | **Std. Beta** |
| --- | --- | --- | --- | --- |
| (Intercept) | -0.004 | [-0.136, 0.127] | 0.947 | 0.073 |
| Recent Life Events | 0.023 | [-0.017, 0.063] | 0.262 | 0.022 |
| Gender (ref: Female) | -0.048 | [-0.128, 0.033] | 0.245 | -0.044 |
| Psychological Resilience | 0 | [-0.040, 0.040] | 0.999 | 0 |
| **SDMT at baseline** | 0.759 | [0.717, 0.801] | <.001 | 0.743 |
| Age | -0.003 | [-0.029, 0.022] | 0.804 | -0.006 |
| Education | 0.023 | [-0.021, 0.067] | 0.300 | 0.027 |
| Depression | -0.026 | [-0.069, 0.017] | 0.240 | -0.026 |
| APOE (4+/4- vs non-carrier) | -0.066 | [-0.154, 0.021] | 0.138 | -0.068 |
| APOE (4+/4+ vs non-carrier) | -0.211 | [-0.563, 0.141] | 0.240 | -0.218 |
| Household Income | 0.01 | [-0.025, 0.044] | 0.576 | -0.001 |
| Hypertension | -0.053 | [-0.130, 0.024] | 0.179 | -0.054 |
| Diabetes | -0.003 | [-0.124, 0.119] | 0.964 | -0.001 |
| Smoking | -0.067 | [-0.252, 0.118] | 0.476 | -0.07 |
| Non-English Speaking Background | -0.061 | [-0.240, 0.118] | 0.502 | -0.067 |
| Exercise (none/mild vs moderate) | -0.026 | [-0.110, 0.058] | 0.545 | -0.027 |
| Exercise (vigorous vs moderate) | 0.106 | [-0.007, 0.220] | 0.066 | 0.109 |

Multiple R-squared: 0.5802; Adjusted R-squared: 0.574; F-statistic: 93.9 on 16 and 1087 DF; p-value: < 2.2e-16

Lifetime trauma and symbol digit modalities test (*n =* 1106)

| **Predictor** | **Beta** | **95% CI** | **p** | **Std. Beta** |
| --- | --- | --- | --- | --- |
| (Intercept) | -0.01 | [-0.141, 0.122] | 0.885 | 0.066 |
| Lifetime Trauma | -0.04 | [-0.080, 0.000] | 0.052 | -0.04 |
| Gender (ref: Female) | -0.032 | [-0.114, 0.051] | 0.452 | -0.028 |
| Psychological Resilience | 0.006 | [-0.034, 0.046] | 0.770 | 0.006 |
| Age | -0.003 | [-0.028, 0.023] | 0.829 | -0.005 |
| **SDMT at baseline** | 0.754 | [0.712, 0.796] | <.001 | 0.738 |
| Education | 0.025 | [-0.018, 0.069] | 0.254 | 0.029 |
| Depression | -0.018 | [-0.061, 0.026] | 0.426 | -0.017 |
| APOE (4+/4- vs non-carrier) | -0.062 | [-0.150, 0.025] | 0.162 | -0.064 |
| APOE (4+/4+ vs non-carrier) | -0.217 | [-0.568, 0.135] | 0.227 | -0.224 |
| Household Income | 0.009 | [-0.025, 0.044] | 0.592 | -0.002 |
| Hypertension | -0.054 | [-0.131, 0.022] | 0.165 | -0.055 |
| Diabetes | 0.004 | [-0.117, 0.125] | 0.950 | 0.006 |
| Smoking | -0.059 | [-0.243, 0.125] | 0.530 | -0.062 |
| Non-English Speaking Background | -0.069 | [-0.246, 0.108] | 0.443 | -0.075 |
| Exercise (none/mild vs moderate) | -0.033 | [-0.118, 0.051] | 0.435 | -0.035 |
| Exercise (vigorous vs moderate) | 0.11 | [-0.003, 0.223] | 0.057 | 0.113 |

Multiple R-squared: 0.5815; Adjusted R-squared: 0.5753; F-statistic: 94.56 on 16 and 1089 DF; p-value: < 2.2e-16

Recent life event and trail making test A (*n =* 1103)

| **Predictor** | **Beta** | **95% CI** | **p** | **Std. Beta** |
| --- | --- | --- | --- | --- |
| (Intercept) | 1.59 | [1.389, 1.791] | <.001 | -0.09 |
| Recent Life Events | -0.001 | [-0.017, 0.015] | 0.904 | -0.003 |
| Gender (ref: Female) | 0.017 | [-0.014, 0.049] | 0.283 | 0.047 |
| Psychological Resilience | -0.007 | [-0.023, 0.009] | 0.376 | -0.023 |
| **Trail Making A at baseline** | 0.578 | [0.523, 0.632] | <.001 | 0.527 |
| Age | 0.001 | [-0.009, 0.011] | 0.852 | 0.008 |
| Education | 0 | [-0.017, 0.017] | 0.972 | -0.013 |
| **Depression** | 0.023 | [0.006, 0.040] | 0.008 | 0.071 |
| APOE (4+/4- vs non-carrier) | 0.002 | [-0.033, 0.037] | 0.910 | 0.003 |
| APOE (4+/4+ vs non-carrier) | -0.034 | [-0.174, 0.106] | 0.630 | -0.113 |
| **Household Income** | -0.024 | [-0.038, -0.010] | <.001 | -0.13 |
| Hypertension | 0.016 | [-0.015, 0.046] | 0.316 | 0.048 |
| Diabetes | 0.045 | [-0.003, 0.093] | 0.065 | 0.145 |
| Smoking | -0.006 | [-0.079, 0.068] | 0.881 | 0.006 |
| **Non-English Speaking Background** | 0.121 | [0.049, 0.192] | <.001 | 0.401 |
| Exercise (none/mild vs moderate) | 0.016 | [-0.017, 0.050] | 0.341 | 0.058 |
| Exercise (vigorous vs moderate) | -0.013 | [-0.058, 0.032] | 0.563 | -0.036 |

Multiple R-squared: 0.3448; Adjusted R-squared: 0.3351; F-statistic: 35.72 on 16 and 1086 DF; p-value: < 2.2e-16

Lifetime trauma and trail making test A (*n =* 1105)

| **Predictor** | **Beta** | **95% CI** | **p** | **Std. Beta** |
| --- | --- | --- | --- | --- |
| (Intercept) | 1.612 | [1.411, 1.813] | <.001 | -0.077 |
| **Lifetime Trauma** | 0.019 | [0.003, 0.035] | 0.021 | 0.059 |
| Gender (ref: Female) | 0.008 | [-0.025, 0.041] | 0.630 | 0.016 |
| Psychological Resilience | -0.01 | [-0.025, 0.006] | 0.239 | -0.031 |
| Age | 0.001 | [-0.009, 0.011] | 0.892 | 0.007 |
| **Trail Making A at baseline** | 0.573 | [0.518, 0.627] | <.001 | 0.523 |
| Education | -0.001 | [-0.018, 0.016] | 0.941 | -0.015 |
| **Depression** | 0.021 | [0.004, 0.038] | 0.017 | 0.065 |
| APOE (4+/4- vs non-carrier) | 0.001 | [-0.033, 0.036] | 0.942 | 0.001 |
| APOE (4+/4+ vs non-carrier) | -0.031 | [-0.170, 0.109] | 0.667 | -0.1 |
| **Household Income** | -0.024 | [-0.038, -0.010] | <.001 | -0.129 |
| Hypertension | 0.017 | [-0.014, 0.047] | 0.285 | 0.051 |
| Diabetes | 0.043 | [-0.005, 0.090] | 0.082 | 0.136 |
| Smoking | -0.007 | [-0.081, 0.066] | 0.845 | 0.001 |
| **Non-English Speaking Background** | 0.118 | [0.047, 0.189] | 0.001 | 0.391 |
| Exercise (none/mild vs moderate) | 0.019 | [-0.015, 0.052] | 0.269 | 0.066 |
| Exercise (vigorous vs moderate) | -0.014 | [-0.059, 0.031] | 0.534 | -0.039 |

Multiple R-squared: 0.3474; Adjusted R-squared: 0.3378; F-statistic: 36.2 on 16 and 1088 DF; p-value: < 2.2e-16

Recent life event and trail making test B (*n =* 1099)

| **Predictor** | **Beta** | **95% CI** | **p** | **Std. Beta** |
| --- | --- | --- | --- | --- |
| (Intercept) | 1.309 | [1.072, 1.546] | <.001 | -0.139 |
| Recent Life Events | 0.004 | [-0.014, 0.022] | 0.685 | 0.009 |
| Gender (ref: Female) | 0.032 | [-0.004, 0.068] | 0.084 | 0.078 |
| Psychological Resilience | 0.001 | [-0.017, 0.019] | 0.895 | 0.003 |
| **Trail Making Test B (baseline)** | 0.715 | [0.663, 0.767] | <.001 | 0.625 |
| Age | 0.008 | [-0.004, 0.019] | 0.174 | 0.031 |
| **Education** | -0.029 | [-0.049, -0.010] | 0.003 | -0.075 |
| **Depression** | 0.049 | [0.029, 0.068] | <.001 | 0.117 |
| APOE (4+/4- vs non-carrier) | 0.02 | [-0.019, 0.059] | 0.321 | 0.051 |
| APOE (4+/4+ vs non-carrier) | -0.008 | [-0.165, 0.150] | 0.923 | -0.019 |
| Household Income | -0.004 | [-0.020, 0.011] | 0.575 | -0.009 |
| Hypertension | 0.028 | [-0.007, 0.063] | 0.111 | 0.071 |
| Diabetes | 0.027 | [-0.027, 0.082] | 0.325 | 0.069 |
| Smoking | -0.021 | [-0.104, 0.062] | 0.619 | -0.051 |
| Non-English Speaking Background | 0.086 | [0.004, 0.168] | 0.041 | 0.222 |
| Exercise (none/mild vs moderate) | 0.012 | [-0.026, 0.050] | 0.530 | 0.032 |
| Exercise (vigorous vs moderate) | 0.049 | [-0.002, 0.099] | 0.061 | 0.124 |

Multiple R-squared: 0.4764; Adjusted R-squared: 0.4687; F-statistic: 61.53 on 16 and 1082 DF; p-value: < 2.2e-16

Lifetime trauma and trail making test B (*n =* 1101)

| **Predictor** | **Beta** | **95% CI** | **p** | **Std. Beta** |
| --- | --- | --- | --- | --- |
| (Intercept) | 1.327 | [1.090, 1.565] | <.001 | -0.13 |
| Lifetime Trauma | 0.015 | [-0.003, 0.033] | 0.108 | 0.037 |
| Gender (ref: Female) | 0.025 | [-0.012, 0.062] | 0.187 | 0.06 |
| Psychological Resilience | 0 | [-0.018, 0.018] | 0.986 | 0 |
| Age | 0.008 | [-0.004, 0.019] | 0.174 | 0.031 |
| **Trail Making Test B (baseline)** | 0.712 | [0.659, 0.764] | <.001 | 0.622 |
| **Education** | -0.03 | [-0.049, -0.010] | 0.003 | -0.077 |
| **Depression** | 0.047 | [0.028, 0.067] | <.001 | 0.114 |
| APOE (4+/4- vs non-carrier) | 0.018 | [-0.021, 0.057] | 0.366 | 0.046 |
| APOE (4+/4+ vs non-carrier) | -0.004 | [-0.161, 0.154] | 0.965 | -0.008 |
| Household Income | -0.005 | [-0.020, 0.011] | 0.566 | -0.008 |
| Hypertension | 0.028 | [-0.006, 0.063] | 0.106 | 0.072 |
| Diabetes | 0.026 | [-0.028, 0.080] | 0.351 | 0.065 |
| Smoking | -0.021 | [-0.104, 0.061] | 0.615 | -0.051 |
| Non-English Speaking Background | 0.087 | [0.006, 0.168] | 0.036 | 0.225 |
| Exercise (none/mild vs moderate) | 0.013 | [-0.025, 0.051] | 0.490 | 0.035 |
| Exercise (vigorous vs moderate) | 0.048 | [-0.002, 0.099] | 0.062 | 0.123 |

Multiple R-squared: 0.4773; Adjusted R-squared: 0.4696; F-statistic: 61.87 on 16 and 1084 DF; p-value: < 2.2e-16

**Supplementary Table 4. Summary of fully adjusted models with interaction terms.**

Recent life events and MCI incidence (*n =* 1195)

| **Predictor** | **OR [95% CI]** | **p** | **Std. Beta** |
| --- | --- | --- | --- |
| (Intercept) | 0.06 [0.03, 0.13] | <.001 | -2.879 |
| Recent Life Events | 1.14 [0.82, 1.57] | 0.438 | 0.124 |
| Gender (ref: Female) | 1.05 [0.66, 1.67] | 0.839 | 0.05 |
| Psychological Resilience | 1.14 [0.83, 1.57] | 0.403 | 0.134 |
| Age | 1.08 [0.93, 1.25] | 0.310 | 0.114 |
| **Education** | 0.71 [0.56, 0.89] | 0.003 | -0.340 |
| **Depression** | 1.35 [1.08, 1.68] | 0.008 | 0.282 |
| **APOE (4+/4- vs non-carrier)** | 1.92 [1.21, 3.06] | 0.006 | 0.654 |
| APOE (4+/4+ vs non-carrier) | 2.59 [0.55, 12.17] | 0.228 | 0.952 |
| Household Income | 1.00 [0.82, 1.23] | 0.969 | 0.005 |
| Hypertension | 0.84 [0.54, 1.32] | 0.449 | -0.174 |
| **Diabetes** | 2.40 [1.38, 4.15] | 0.002 | 0.875 |
| Smoking | 1.43 [0.56, 3.67] | 0.455 | 0.359 |
| Non-English Speaking Background | 1.68 [0.68, 4.15] | 0.264 | 0.516 |
| Exercise (none/mild vs moderate) | 0.74 [0.45, 1.22] | 0.240 | -0.300 |
| Exercise (vigorous vs moderate) | 1.43 [0.76, 2.68] | 0.265 | 0.357 |
| Recent Life Events:Gender (ref: Female) | 0.77 [0.48, 1.25] | 0.294 | -0.252 |
| Recent Life Events:Psychological Resilience | 1.01 [0.79, 1.31] | 0.923 | 0.012 |
| Gender (ref: Female):Psychological Resilience | 0.89 [0.57, 1.39] | 0.605 | -0.114 |
| Recent Life Events:Gender (ref: Female):Psychological Resilience | 0.86 [0.52, 1.41] | 0.542 | -0.15 |

Multiple R-squared: 0.0004117; Adjusted R-squared: -0.01575; F-statistic: 0.02547 on 19 and 1175 DF; p-value: 1

Lifetime Trauma and MCI incidence (*n =* 1197)

| **Predictor** | **OR [95% CI]** | **p** | **Std. Beta** |
| --- | --- | --- | --- |
| (Intercept) | 0.06 [0.03, 0.13] | <.001 | -2.811 |
| **Lifetime Trauma** | 1.96 [1.41, 2.73] | <.001 | 0.67 |
| Gender (ref: Female) | 0.75 [0.45, 1.26] | 0.276 | -0.311 |
| Psychological Resilience | 1.08 [0.78, 1.49] | 0.652 | 0.073 |
| Age | 1.08 [0.93, 1.26] | 0.301 | 0.12 |
| **Education** | 0.70 [0.55, 0.88] | 0.003 | -0.368 |
| Depression | 1.22 [0.97, 1.53] | 0.089 | 0.188 |
| **APOE (4+/4- vs non-carrier)** | 1.77 [1.10, 2.85] | 0.019 | 0.579 |
| APOE (4+/4+ vs non-carrier) | 2.56 [0.52, 12.57] | 0.248 | 0.956 |
| Household Income | 1.01 [0.82, 1.23] | 0.953 | 0.141 |
| Hypertension | 0.88 [0.56, 1.40] | 0.603 | -0.123 |
| **Diabetes** | 2.31 [1.30, 4.09] | 0.004 | 0.826 |
| Smoking | 1.40 [0.53, 3.68] | 0.493 | 0.322 |
| Non-English Speaking Background | 1.73 [0.67, 4.44] | 0.256 | 0.555 |
| Exercise (none/mild vs moderate) | 0.75 [0.45, 1.27] | 0.285 | -0.279 |
| Exercise (vigorous vs moderate) | 1.45 [0.76, 2.75] | 0.255 | 0.366 |
| Lifetime Trauma:Gender (ref: Female) | 0.86 [0.57, 1.31] | 0.49 | -0.148 |
| Lifetime Trauma:Psychological Resilience | 0.92 [0.67, 1.26] | 0.595 | -0.087 |
| Gender (ref: Female):Psychological Resilience | 0.94 [0.57, 1.55] | 0.795 | -0.06 |
| Lifetime Trauma:Gender (ref: Female):Psychological Resilience | 0.98 [0.65, 1.47] | 0.924 | -0.017 |

Multiple R-squared: 0.001038; Adjusted R-squared: -0.01509; F-statistic: 0.06434 on 19 and 1177 DF; p-value: 1

Recent life events and dementia incidence (*n =* 1195)

| **Predictor** | **OR [95% CI]** | **p** | **Std. Beta** |
| --- | --- | --- | --- |
| (Intercept) | 0.00 [0.00, 0.04] | <.001 | -6.438 |
| Recent Life Events | 0.97 [0.34, 2.83] | 0.961 | -0.011 |
| Gender (ref: Female) | 0.43 [0.03, 7.14] | 0.559 | -0.807 |
| Psychological Resilience | 0.64 [0.27, 1.49] | 0.299 | -0.523 |
| Age | 1.45 [0.95, 2.20] | 0.083 | 0.576 |
| Education | 1.91 [0.94, 3.88] | 0.072 | 0.669 |
| **Depression** | 1.74 [1.00, 3.02] | 0.048 | 0.494 |
| APOE (4+/4- vs non-carrier) | 3.11 [0.88, 10.99] | 0.078 | 1.131 |
| **APOE (4+/4+ vs non-carrier)** | 26.00 [3.77, 179.40] | <.001 | 3.248 |
| Household Income | 0.77 [0.43, 1.38] | 0.38 | -1.238 |
| Hypertension | 1.17 [0.34, 4.06] | 0.801 | 0.202 |
| Diabetes | 1.99 [0.45, 8.86] | 0.367 | 0.78 |
| Smoking | 0.00 [0.00, Inf] | 0.994 | -16.226 |
| Non-English Speaking Background | 0.00 [0.00, Inf] | 0.994 | -15.922 |
| Exercise (none/mild vs moderate) | 2.17 [0.55, 8.54] | 0.269 | 0.8 |
| Exercise (vigorous vs moderate) | 0.94 [0.10, 9.19] | 0.956 | 0.058 |
| Recent Life Events:Gender (ref: Female) | 0.07 [0.00, 4.13] | 0.197 | -2.775 |
| Recent Life Events:Psychological Resilience | 1.80 [0.73, 4.45] | 0.201 | 0.563 |
| Gender (ref: Female):Psychological Resilience | 0.86 [0.11, 6.84] | 0.884 | -0.068 |
| Recent Life Events:Gender (ref: Female):Psychological Resilience | 0.20 [0.01, 3.95] | 0.292 | -1.583 |

Multiple R-squared: 0.0001944; Adjusted R-squared: -0.01597; F-statistic: 0.01203 on 19 and 1175 DF; p-value: 1

Lifetime trauma and dementia incidence (*n =* 1197)

| **Predictor** | **OR [95% CI]** | **p** | **Std. Beta** |
| --- | --- | --- | --- |
| (Intercept) | 0.00 [0.00, 0.04] | <.001 | -6.258 |
| **Lifetime Trauma** | 2.42 [1.11, 5.27] | 0.026 | 0.906 |
| Gender (ref: Female) | 0.91 [0.21, 4.01] | 0.902 | -0.051 |
| Psychological Resilience | 0.68 [0.29, 1.63] | 0.392 | -0.433 |
| Age | 1.35 [0.91, 2.01] | 0.141 | 0.451 |
| Education | 1.79 [0.89, 3.58] | 0.100 | 0.601 |
| Depression | 1.40 [0.81, 2.43] | 0.233 | 0.302 |
| APOE (4+/4- vs non-carrier) | 3.16 [0.89, 11.18] | 0.075 | 1.136 |
| **APOE (4+/4+ vs non-carrier)** | 29.63 [4.37, 201.16] | <.001 | 3.342 |
| Household Income | 0.81 [0.45, 1.44] | 0.468 | -1.124 |
| Hypertension | 1.24 [0.35, 4.35] | 0.741 | 0.218 |
| Diabetes | 1.81 [0.39, 8.31] | 0.448 | 0.647 |
| Smoking | 0.00 [0.00, Inf] | 0.994 | -15.947 |
| Non-English Speaking Background | 0.00 [0.00, Inf] | 0.994 | -16.139 |
| Exercise (none/mild vs moderate) | 2.33 [0.59, 9.12] | 0.226 | 0.895 |
| Exercise (vigorous vs moderate) | 0.85 [0.09, 8.24] | 0.89 | -0.116 |
| Lifetime Trauma:Gender (ref: Female) | 0.66 [0.24, 1.81] | 0.418 | -0.441 |
| Lifetime Trauma:Psychological Resilience | 1.00 [0.50, 2.00] | 0.99 | -0.007 |
| Gender (ref: Female):Psychological Resilience | 1.04 [0.28, 3.90] | 0.959 | 0.083 |
| Lifetime Trauma:Gender (ref: Female):Psychological Resilience | 1.17 [0.45, 3.05] | 0.744 | 0.165 |

Multiple R-squared: 0.0002037; Adjusted R-squared: -0.01594; F-statistic: 0.01262 on 19 and 1177 DF; p-value: 1

Recent life events and MMSE (*n =* 1105)

| **Predictor** | **Beta** | **95% CI** | **p** | **Std. Beta** |
| --- | --- | --- | --- | --- |
| (Intercept) | 0.108 | [-0.044, 0.261] | 0.165 | 0.131 |
| Recent Life Events | -0.008 | [-0.078, 0.063] | 0.833 | -0.01 |
| **Gender (ref: Female)** | -0.265 | [-0.358, -0.172] | <.001 | -0.326 |
| Psychological Resilience | 0.018 | [-0.046, 0.082] | 0.575 | 0.023 |
| **MMSE at baseline** | 0.285 | [0.229, 0.341] | <.001 | 0.290 |
| Age | -0.019 | [-0.049, 0.011] | 0.205 | -0.036 |
| **Education** | 0.135 | [0.084, 0.186] | <.001 | 0.164 |
| Depression | -0.047 | [-0.097, 0.003] | 0.067 | -0.054 |
| APOE (4+/4- vs non-carrier) | 0.03 | [-0.071, 0.132] | 0.559 | 0.038 |
| APOE (4+/4+ vs non-carrier) | -0.057 | [-0.464, 0.350] | 0.783 | -0.07 |
| Household Income | 0.013 | [-0.027, 0.053] | 0.523 | 0.028 |
| **Hypertension** | 0.101 | [0.012, 0.190] | 0.027 | 0.125 |
| Diabetes | -0.067 | [-0.207, 0.072] | 0.344 | -0.083 |
| Smoking | -0.019 | [-0.236, 0.198] | 0.865 | -0.028 |
| Non-English Speaking Background | -0.199 | [-0.408, 0.010] | 0.062 | -0.249 |
| Exercise (none/mild vs moderate) | -0.033 | [-0.130, 0.065] | 0.514 | -0.042 |
| Exercise (vigorous vs moderate) | 0.012 | [-0.119, 0.143] | 0.855 | 0.014 |
| Recent Life Events:Gender (ref: Female) | -0.057 | [-0.150, 0.037] | 0.236 | -0.066 |
| Recent Life Events:Psychological Resilience | -0.003 | [-0.064, 0.058] | 0.919 | -0.004 |
| Gender (ref: Female):Psychological Resilience | -0.03 | [-0.121, 0.060] | 0.513 | -0.038 |
| Recent Life Events:Gender (ref: Female):Psychological Resilience | 0.036 | [-0.064, 0.136] | 0.481 | 0.042 |

Multiple R-squared: 0.1807; Adjusted R-squared: 0.1656; F-statistic: 11.96 on 20 and 1084 DF; p-value: < 2.2e-16

Lifetime trauma and MMSE (*n =* 1107)

| **Predictor** | **Beta** | **95% CI** | **p** | **Std. Beta** |
| --- | --- | --- | --- | --- |
| (Intercept) | 0.113 | [-0.042, 0.267] | 0.153 | 0.135 |
| Lifetime Trauma | -0.039 | [-0.125, 0.047] | 0.373 | -0.048 |
| **Gender (ref: Female)** | -0.25 | [-0.349, -0.152] | <.001 | -0.308 |
| Psychological Resilience | 0.008 | [-0.062, 0.078] | 0.827 | 0.01 |
| Age | -0.02 | [-0.050, 0.010] | 0.184 | -0.038 |
| **MMSE at baseline** | 0.292 | [0.236, 0.347] | <.001 | 0.294 |
| **Education** | 0.13 | [0.078, 0.181] | <.001 | 0.155 |
| Depression | -0.048 | [-0.099, 0.002] | 0.061 | -0.055 |
| APOE (4+/4- vs non-carrier) | 0.037 | [-0.065, 0.140] | 0.478 | 0.046 |
| APOE (4+/4+ vs non-carrier) | -0.053 | [-0.463, 0.357] | 0.799 | -0.064 |
| Household Income | 0.012 | [-0.028, 0.052] | 0.566 | 0.031 |
| Hypertension | 0.087 | [-0.002, 0.177] | 0.056 | 0.108 |
| Diabetes | -0.062 | [-0.203, 0.079] | 0.388 | -0.077 |
| Smoking | -0.011 | [-0.229, 0.207] | 0.922 | -0.018 |
| **Non-English Speaking Background** | -0.248 | [-0.457, -0.039] | 0.02 | -0.306 |
| Exercise (none/mild vs moderate) | -0.043 | [-0.142, 0.055] | 0.388 | -0.054 |
| Exercise (vigorous vs moderate) | 0.013 | [-0.119, 0.144] | 0.851 | 0.014 |
| Lifetime Trauma:Gender (ref: Female) | 0.036 | [-0.066, 0.139] | 0.489 | 0.044 |
| Lifetime Trauma:Psychological Resilience | -0.038 | [-0.126, 0.051] | 0.407 | -0.044 |
| Gender (ref: Female):Psychological Resilience | -0.026 | [-0.122, 0.070] | 0.596 | -0.031 |
| Lifetime Trauma:Gender (ref: Female):Psychological Resilience | 0.015 | [-0.091, 0.121] | 0.783 | 0.017 |

Multiple R-squared: 0.1825; Adjusted R-squared: 0.1675; F-statistic: 12.13 on 20 and 1086 DF; p-value: < 2.2e-16

Recent life events and immediate recall (*n =* 1183)

| **Predictor** | **Beta** | **95% CI** | **p** | **Std. Beta** |
| --- | --- | --- | --- | --- |
| (Intercept) | 0.004 | [-0.167, 0.175] | 0.963 | 0.14 |
| Recent Life Events | -0.023 | [-0.102, 0.056] | 0.568 | -0.023 |
| **Gender (ref: Female)** | -0.153 | [-0.260, -0.046] | 0.005 | -0.15 |
| Psychological Resilience | 0.003 | [-0.068, 0.074] | 0.937 | 0.004 |
| **Immediate recall at baseline** | 0.405 | [0.352, 0.459] | <.001 | 0.407 |
| **Age** | -0.056 | [-0.089, -0.023] | <.001 | -0.086 |
| **Education** | 0.154 | [0.099, 0.210] | <.001 | 0.158 |
| Depression | -0.012 | [-0.067, 0.044] | 0.682 | -0.011 |
| APOE (4+/4- vs non-carrier) | -0.107 | [-0.221, 0.006] | 0.063 | -0.107 |
| APOE (4+/4+ vs non-carrier) | -0.193 | [-0.617, 0.230] | 0.370 | -0.193 |
| Household Income | 0.037 | [-0.008, 0.082] | 0.105 | 0.069 |
| Hypertension | 0.022 | [-0.077, 0.121] | 0.664 | 0.023 |
| Diabetes | -0.053 | [-0.208, 0.102] | 0.501 | -0.055 |
| Smoking | -0.056 | [-0.303, 0.192] | 0.659 | -0.069 |
| Non-English Speaking Background | -0.174 | [-0.409, 0.061] | 0.146 | -0.182 |
| Exercise (none/mild vs moderate) | 0.006 | [-0.103, 0.116] | 0.907 | 0.003 |
| **Exercise (vigorous vs moderate)** | -0.176 | [-0.322, -0.031] | 0.018 | -0.184 |
| Recent Life Events:Gender (ref: Female) | 0.037 | [-0.067, 0.142] | 0.484 | 0.037 |
| Recent Life Events:Psychological Resilience | 0.034 | [-0.034, 0.102] | 0.332 | 0.032 |
| Gender (ref: Female):Psychological Resilience | 0.052 | [-0.048, 0.151] | 0.308 | 0.052 |
| Recent Life Events:Gender (ref: Female):Psychological Resilience | -0.083 | [-0.189, 0.022] | 0.121 | -0.083 |

Multiple R-squared: 0.2618; Adjusted R-squared: 0.2491; F-statistic: 20.61 on 20 and 1162 DF; p-value: < 2.2e-16

Lifetime trauma and immediate recall (*n =* 1185)

| **Predictor** | **Beta** | **95% CI** | **p** | **Std. Beta** |
| --- | --- | --- | --- | --- |
| (Intercept) | 0.011 | [-0.162, 0.183] | 0.903 | 0.139 |
| Lifetime Trauma | 0.007 | [-0.084, 0.098] | 0.879 | 0.006 |
| Gender (ref: Female) | -0.153 | [-0.264, -0.041] | 0.007 | -0.149 |
| Psychological Resilience | 0.006 | [-0.069, 0.081] | 0.879 | 0.008 |
| **Age** | -0.057 | [-0.091, -0.024] | <.001 | -0.088 |
| **Immediate recall at baseline** | 0.407 | [0.354, 0.461] | <.001 | 0.406 |
| **Education** | 0.157 | [0.101, 0.213] | <.001 | 0.16 |
| Depression | -0.008 | [-0.063, 0.048] | 0.786 | -0.008 |
| APOE (4+/4- vs non-carrier) | -0.095 | [-0.208, 0.019] | 0.103 | -0.094 |
| APOE (4+/4+ vs non-carrier) | -0.199 | [-0.624, 0.226] | 0.359 | -0.199 |
| Household Income | 0.035 | [-0.010, 0.080] | 0.124 | 0.065 |
| Hypertension | 0.026 | [-0.073, 0.126] | 0.603 | 0.027 |
| Diabetes | -0.053 | [-0.209, 0.102] | 0.501 | -0.055 |
| Smoking | -0.067 | [-0.315, 0.180] | 0.594 | -0.08 |
| Non-English Speaking Background | -0.194 | [-0.428, 0.040] | 0.105 | -0.2 |
| Exercise (none/mild vs moderate) | 0.006 | [-0.104, 0.115] | 0.915 | 0.003 |
| **Exercise (vigorous vs moderate)** | -0.175 | [-0.321, -0.028] | 0.019 | -0.181 |
| Lifetime Trauma:Gender (ref: Female) | -0.043 | [-0.153, 0.067] | 0.447 | -0.042 |
| Lifetime Trauma:Psychological Resilience | 0.011 | [-0.079, 0.101] | 0.81 | 0.012 |
| Gender (ref: Female):Psychological Resilience | 0.064 | [-0.040, 0.167] | 0.229 | 0.062 |
| Lifetime Trauma:Gender (ref: Female):Psychological Resilience | -0.032 | [-0.142, 0.078] | 0.565 | -0.034 |

Multiple R-squared: 0.2629; Adjusted R-squared: 0.2502; F-statistic: 20.75 on 20 and 1164 DF; p-value: < 2.2e-16

Recent life events and digit span backward (*n =* 1151)

| **Predictor** | **Beta** | **95% CI** | **p** | **Std. Beta** |
| --- | --- | --- | --- | --- |
| (Intercept) | 0.005 | [-0.161, 0.171] | 0.951 | 0.009 |
| Recent Life Events | 0.032 | [-0.045, 0.108] | 0.415 | 0.032 |
| Gender (ref: Female) | 0.053 | [-0.047, 0.153] | 0.296 | 0.048 |
| Psychological Resilience | 0.017 | [-0.053, 0.087] | 0.641 | 0.016 |
| **Digit span backward at baseline** | 0.546 | [0.497, 0.595] | <.001 | 0.552 |
| **Age** | -0.035 | [-0.067, -0.003] | 0.033 | -0.051 |
| Education | 0.018 | [-0.036, 0.072] | 0.508 | 0.015 |
| **Depression** | -0.065 | [-0.119, -0.011] | 0.019 | -0.061 |
| APOE (4+/4- vs non-carrier) | -0.033 | [-0.143, 0.077] | 0.553 | -0.034 |
| APOE (4+/4+ vs non-carrier) | 0.211 | [-0.193, 0.616] | 0.305 | 0.212 |
| Household Income | -0.01 | [-0.053, 0.033] | 0.642 | -0.008 |
| Hypertension | 0 | [-0.096, 0.096] | 1 | 0 |
| Diabetes | -0.076 | [-0.224, 0.073] | 0.316 | -0.077 |
| Smoking | -0.057 | [-0.290, 0.177] | 0.634 | -0.054 |
| **Non-English Speaking Background** | -0.332 | [-0.559, -0.105] | 0.004 | -0.33 |
| Exercise (none/mild vs moderate) | 0.002 | [-0.103, 0.108] | 0.969 | 0.003 |
| Exercise (vigorous vs moderate) | -0.049 | [-0.190, 0.092] | 0.497 | -0.048 |
| Recent Life Events:Gender (ref: Female) | -0.027 | [-0.128, 0.075] | 0.605 | -0.027 |
| Recent Life Events:Psychological Resilience | 0.02 | [-0.047, 0.087] | 0.563 | 0.019 |
| **Gender (ref: Female):Psychological Resilience** | -0.137 | [-0.235, -0.040] | 0.006 | -0.134 |
| Recent Life Events:Gender (ref: Female):Psychological Resilience | -0.023 | [-0.126, 0.080] | 0.657 | -0.022 |

Multiple R-squared: 0.352; Adjusted R-squared: 0.3405; F-statistic: 30.69 on 20 and 1130 DF; p-value: < 2.2e-16

Lifetime trauma and digit span backward (*n =* 1153)

| **Predictor** | **Beta** | **95% CI** | **p** | **Std. Beta** |
| --- | --- | --- | --- | --- |
| (Intercept) | -0.021 | [-0.186, 0.145] | 0.806 | -0.017 |
| Lifetime Trauma | -0.107 | [-0.196, -0.018] | 0.018 | -0.107 |
| Gender (ref: Female) | 0.069 | [-0.035, 0.172] | 0.194 | 0.063 |
| Psychological Resilience | 0.014 | [-0.061, 0.088] | 0.715 | 0.013 |
| **Age** | -0.035 | [-0.066, -0.003] | 0.031 | -0.051 |
| **Digit span backward at baseline** | 0.544 | [0.495, 0.593] | <.001 | 0.55 |
| Education | 0.017 | [-0.037, 0.070] | 0.548 | 0.013 |
| **Depression** | -0.056 | [-0.109, -0.002] | 0.043 | -0.052 |
| APOE (4+/4- vs non-carrier) | -0.019 | [-0.128, 0.091] | 0.737 | -0.019 |
| APOE (4+/4+ vs non-carrier) | 0.224 | [-0.178, 0.626] | 0.275 | 0.225 |
| Household Income | -0.01 | [-0.053, 0.033] | 0.656 | -0.005 |
| Hypertension | -0.003 | [-0.099, 0.092] | 0.948 | -0.003 |
| Diabetes | -0.073 | [-0.221, 0.075] | 0.333 | -0.074 |
| Smoking | -0.07 | [-0.302, 0.161] | 0.552 | -0.068 |
| **Non-English Speaking Background** | -0.322 | [-0.546, -0.098] | 0.005 | -0.319 |
| Exercise (none/mild vs moderate) | -0.001 | [-0.106, 0.104] | 0.987 | 0 |
| Exercise (vigorous vs moderate) | -0.05 | [-0.191, 0.090] | 0.481 | -0.05 |
| Lifetime Trauma:Gender (ref: Female) | 0.103 | [-0.003, 0.210] | 0.058 | 0.104 |
| Lifetime Trauma:Psychological Resilience | -0.038 | [-0.128, 0.051] | 0.404 | -0.038 |
| **Gender (ref: Female):Psychological Resilience** | -0.151 | [-0.252, -0.050] | 0.003 | -0.146 |
| Lifetime Trauma:Gender (ref: Female):Psychological Resilience | 0.113 | [0.005, 0.222] | 0.04 | 0.112 |

Multiple R-squared: 0.3594; Adjusted R-squared: 0.3481; F-statistic: 31.76 on 20 and 1132 DF; p-value: < 2.2e-16

Recent life event and symbol digit modalities test (*n =* 1104)

| **Predictor** | **Beta** | **95% CI** | **p** | **Std. Beta** |
| --- | --- | --- | --- | --- |
| (Intercept) | -0.004 | [-0.136, 0.127] | 0.947 | 0.075 |
| Recent Life Events | 0.022 | [-0.039, 0.083] | 0.487 | 0.021 |
| Gender (ref: Female) | -0.049 | [-0.129, 0.032] | 0.233 | -0.047 |
| Psychological Resilience | 0.028 | [-0.027, 0.084] | 0.316 | 0.028 |
| **SDMT at baseline** | 0.757 | [0.714, 0.799] | <.001 | 0.741 |
| Age | -0.003 | [-0.028, 0.023] | 0.842 | -0.005 |
| Education | 0.021 | [-0.023, 0.065] | 0.343 | 0.025 |
| Depression | -0.025 | [-0.068, 0.019] | 0.264 | -0.024 |
| APOE (4+/4- vs non-carrier) | -0.069 | [-0.157, 0.019] | 0.123 | -0.071 |
| APOE (4+/4+ vs non-carrier) | -0.222 | [-0.574, 0.131] | 0.217 | -0.229 |
| Household Income | 0.01 | [-0.024, 0.045] | 0.555 | -0.001 |
| Hypertension | -0.053 | [-0.130, 0.024] | 0.178 | -0.054 |
| Diabetes | -0.01 | [-0.132, 0.112] | 0.871 | -0.008 |
| Smoking | -0.068 | [-0.254, 0.117] | 0.470 | -0.072 |
| Non-English Speaking Background | -0.063 | [-0.242, 0.116] | 0.493 | -0.068 |
| Exercise (none/mild vs moderate) | -0.03 | [-0.115, 0.055] | 0.486 | -0.032 |
| Exercise (vigorous vs moderate) | 0.107 | [-0.007, 0.221] | 0.065 | 0.11 |
| Recent Life Events:Gender (ref: Female) | -0.002 | [-0.083, 0.079] | 0.969 | -0.001 |
| Recent Life Events:Psychological Resilience | 0.011 | [-0.042, 0.064] | 0.687 | 0.01 |
| Gender (ref: Female):Psychological Resilience | -0.056 | [-0.134, 0.023] | 0.164 | -0.056 |
| Recent Life Events:Gender (ref: Female):Psychological Resilience | 0.025 | [-0.061, 0.112] | 0.562 | 0.025 |

Multiple R-squared: 0.5816; Adjusted R-squared: 0.5738; F-statistic: 75.26 on 20 and 1083 DF; p-value: < 2.2e-16

Lifetime trauma and symbol digit modalities test (*n =* 1106)

| **Predictor** | **Beta** | **95% CI** | **p** | **Std. Beta** |
| --- | --- | --- | --- | --- |
| (Intercept) | -0.005 | [-0.137, 0.127] | 0.942 | 0.071 |
| Lifetime Trauma | -0.033 | [-0.105, 0.038] | 0.362 | -0.034 |
| Gender (ref: Female) | -0.029 | [-0.112, 0.054] | 0.493 | -0.026 |
| Psychological Resilience | 0.029 | [-0.030, 0.088] | 0.331 | 0.029 |
| Age | -0.003 | [-0.028, 0.023] | 0.823 | -0.005 |
| **SDMT at baseline** | 0.753 | [0.711, 0.795] | <.001 | 0.737 |
| Education | 0.025 | [-0.019, 0.069] | 0.268 | 0.029 |
| Depression | -0.018 | [-0.061, 0.025] | 0.415 | -0.018 |
| APOE (4+/4- vs non-carrier) | -0.065 | [-0.153, 0.023] | 0.147 | -0.066 |
| APOE (4+/4+ vs non-carrier) | -0.226 | [-0.578, 0.126] | 0.208 | -0.233 |
| Household Income | 0.009 | [-0.025, 0.044] | 0.602 | -0.002 |
| Hypertension | -0.057 | [-0.134, 0.020] | 0.146 | -0.058 |
| Diabetes | 0 | [-0.121, 0.122] | 0.996 | 0.002 |
| Smoking | -0.057 | [-0.242, 0.128] | 0.544 | -0.06 |
| Non-English Speaking Background | -0.069 | [-0.247, 0.108] | 0.443 | -0.075 |
| Exercise (none/mild vs moderate) | -0.037 | [-0.121, 0.047] | 0.389 | -0.039 |
| Exercise (vigorous vs moderate) | 0.112 | [-0.001, 0.225] | 0.052 | 0.115 |
| Lifetime Trauma:Gender (ref: Female) | -0.007 | [-0.093, 0.080] | 0.882 | -0.006 |
| Lifetime Trauma:Psychological Resilience | -0.009 | [-0.081, 0.064] | 0.812 | -0.008 |
| Gender (ref: Female):Psychological Resilience | -0.044 | [-0.125, 0.037] | 0.290 | -0.044 |
| Lifetime Trauma:Gender (ref: Female):Psychological Resilience | -0.02 | [-0.108, 0.067] | 0.647 | -0.022 |

Multiple R-squared: 0.5827; Adjusted R-squared: 0.575; F-statistic: 75.76 on 20 and 1085 DF; p-value: < 2.2e-16

Recent life event and trail making test A (*n =* 1103)

| **Predictor** | **Beta** | **95% CI** | **p** | **Std. Beta** |
| --- | --- | --- | --- | --- |
| (Intercept) | 1.586 | [1.385, 1.787] | <.001 | -0.089 |
| Recent Life Events | -0.013 | [-0.037, 0.012] | 0.304 | -0.037 |
| Gender (ref: Female) | 0.017 | [-0.015, 0.049] | 0.296 | 0.046 |
| Psychological Resilience | -0.024 | [-0.046, -0.002] | 0.034 | -0.077 |
| **Trail Making A at baseline** | 0.579 | [0.524, 0.633] | <.001 | 0.528 |
| Age | 0.001 | [-0.009, 0.011] | 0.812 | 0.009 |
| Education | 0.001 | [-0.017, 0.018] | 0.949 | -0.011 |
| **Depression** | 0.023 | [0.006, 0.040] | 0.01 | 0.07 |
| APOE (4+/4- vs non-carrier) | 0.002 | [-0.032, 0.037] | 0.891 | 0.004 |
| APOE (4+/4+ vs non-carrier) | -0.028 | [-0.168, 0.112] | 0.695 | -0.092 |
| **Household Income** | -0.024 | [-0.037, -0.010] | <.001 | -0.129 |
| Hypertension | 0.016 | [-0.015, 0.047] | 0.312 | 0.049 |
| Diabetes | 0.048 | [-0.001, 0.096] | 0.053 | 0.152 |
| Smoking | 0.003 | [-0.071, 0.077] | 0.932 | 0.034 |
| **Non-English Speaking Background** | 0.119 | [0.047, 0.191] | 0.001 | 0.395 |
| Exercise (none/mild vs moderate) | 0.017 | [-0.017, 0.051] | 0.319 | 0.061 |
| Exercise (vigorous vs moderate) | -0.014 | [-0.059, 0.031] | 0.538 | -0.039 |
| Recent Life Events:Gender (ref: Female) | 0.021 | [-0.011, 0.053] | 0.194 | 0.063 |
| Recent Life Events:Psychological Resilience | 0.011 | [-0.010, 0.032] | 0.302 | 0.035 |
| **Gender (ref: Female):Psychological Resilience** | 0.031 | [0.000, 0.062] | 0.049 | 0.1 |
| Recent Life Events:Gender (ref: Female):Psychological Resilience | -0.019 | [-0.053, 0.016] | 0.287 | -0.055 |

Multiple R-squared: 0.349; Adjusted R-squared: 0.337; F-statistic: 29.01 on 20 and 1082 DF; p-value: < 2.2e-16

Lifetime trauma and trail making test A (*n =* 1105)

| **Predictor** | **Beta** | **95% CI** | **p** | **Std. Beta** |
| --- | --- | --- | --- | --- |
| (Intercept) | 1.621 | [1.420, 1.822] | <.001 | -0.061 |
| **Lifetime Trauma** | 0.039 | [0.011, 0.067] | 0.007 | 0.123 |
| Gender (ref: Female) | 0.003 | [-0.030, 0.036] | 0.865 | 0.002 |
| **Psychological Resilience** | -0.028 | [-0.051, -0.005] | 0.019 | -0.09 |
| Age | 0.001 | [-0.010, 0.011] | 0.903 | 0.006 |
| **Trail Making A at baseline** | 0.571 | [0.516, 0.626] | <.001 | 0.521 |
| Education | -0.001 | [-0.018, 0.016] | 0.95 | -0.014 |
| **Depression** | 0.02 | [0.003, 0.037] | 0.019 | 0.063 |
| APOE (4+/4- vs non-carrier) | 0.001 | [-0.034, 0.036] | 0.966 | -0.001 |
| APOE (4+/4+ vs non-carrier) | -0.027 | [-0.167, 0.112] | 0.703 | -0.089 |
| **Household Income** | -0.023 | [-0.037, -0.010] | <.001 | -0.126 |
| Hypertension | 0.019 | [-0.012, 0.049] | 0.235 | 0.057 |
| Diabetes | 0.047 | [-0.001, 0.095] | 0.057 | 0.15 |
| Smoking | -0.006 | [-0.079, 0.068] | 0.879 | 0.005 |
| **Non-English Speaking Background** | 0.115 | [0.044, 0.185] | 0.002 | 0.381 |
| Exercise (none/mild vs moderate) | 0.021 | [-0.013, 0.054] | 0.226 | 0.072 |
| Exercise (vigorous vs moderate) | -0.015 | [-0.060, 0.030] | 0.515 | -0.041 |
| Lifetime Trauma:Gender (ref: Female) | -0.03 | [-0.064, 0.004] | 0.088 | -0.096 |
| Lifetime Trauma:Psychological Resilience | -0.009 | [-0.038, 0.019] | 0.52 | -0.032 |
| **Gender (ref: Female):Psychological Resilience** | 0.034 | [0.002, 0.066] | 0.039 | 0.108 |
| Lifetime Trauma:Gender (ref: Female):Psychological Resilience | 0.013 | [-0.021, 0.048] | 0.454 | 0.045 |

Multiple R-squared: 0.3515; Adjusted R-squared: 0.3396; F-statistic: 29.38 on 20 and 1084 DF; p-value: < 2.2e-16

Recent life event and trail making test B (*n =* 1099)

| **Predictor** | **Beta** | **95% CI** | **p** | **Std. Beta** |
| --- | --- | --- | --- | --- |
| (Intercept) | 1.302 | [1.065, 1.539] | <.001 | -0.136 |
| Recent Life Events | -0.01 | [-0.037, 0.017] | 0.481 | -0.023 |
| Gender (ref: Female) | 0.031 | [-0.005, 0.067] | 0.096 | 0.075 |
| Psychological Resilience | -0.004 | [-0.029, 0.021] | 0.750 | -0.011 |
| **Trail Making Test B (baseline)** | 0.717 | [0.665, 0.769] | <.001 | 0.626 |
| Age | 0.009 | [-0.003, 0.020] | 0.142 | 0.033 |
| **Education** | -0.029 | [-0.049, -0.010] | 0.003 | -0.075 |
| **Depression** | 0.049 | [0.030, 0.069] | <.001 | 0.118 |
| APOE (4+/4- vs non-carrier) | 0.019 | [-0.020, 0.059] | 0.340 | 0.049 |
| APOE (4+/4+ vs non-carrier) | -0.006 | [-0.164, 0.151] | 0.936 | -0.016 |
| Household Income | -0.004 | [-0.019, 0.011] | 0.606 | -0.008 |
| Hypertension | 0.028 | [-0.006, 0.063] | 0.110 | 0.072 |
| Diabetes | 0.026 | [-0.028, 0.081] | 0.340 | 0.067 |
| Smoking | -0.012 | [-0.095, 0.071] | 0.781 | -0.028 |
| **Non-English Speaking Background** | 0.084 | [0.001, 0.166] | 0.046 | 0.216 |
| Exercise (none/mild vs moderate) | 0.011 | [-0.027, 0.049] | 0.578 | 0.028 |
| Exercise (vigorous vs moderate) | 0.048 | [-0.003, 0.098] | 0.065 | 0.122 |
| Recent Life Events:Gender (ref: Female) | 0.022 | [-0.014, 0.059] | 0.228 | 0.054 |
| Recent Life Events:Psychological Resilience | 0.018 | [-0.005, 0.042] | 0.127 | 0.045 |
| Gender (ref: Female):Psychological Resilience | 0.009 | [-0.026, 0.044] | 0.615 | 0.023 |
| Recent Life Events:Gender (ref: Female):Psychological Resilience | -0.006 | [-0.045, 0.033] | 0.763 | -0.014 |

Multiple R-squared: 0.479; Adjusted R-squared: 0.4693; F-statistic: 49.55 on 20 and 1078 DF; p-value: < 2.2e-16

Lifetime trauma and trail making test B (*n =* 1101)

| **Predictor** | **Beta** | **95% CI** | **p** | **Std. Beta** |
| --- | --- | --- | --- | --- |
| (Intercept) | 1.326 | [1.088, 1.565] | <.001 | -0.133 |
| Lifetime Trauma | 0.016 | [-0.016, 0.049] | 0.318 | 0.042 |
| Gender (ref: Female) | 0.024 | [-0.013, 0.062] | 0.2 | 0.059 |
| Psychological Resilience | 0.001 | [-0.026, 0.027] | 0.954 | 0.002 |
| Age | 0.008 | [-0.003, 0.019] | 0.169 | 0.031 |
| Trail Making Test B (baseline) | 0.712 | [0.659, 0.764] | <.001 | 0.622 |
| Education | -0.03 | [-0.050, -0.011] | 0.003 | -0.078 |
| Depression | 0.048 | [0.028, 0.067] | <.001 | 0.114 |
| APOE (4+/4- vs non-carrier) | 0.018 | [-0.021, 0.058] | 0.361 | 0.047 |
| APOE (4+/4+ vs non-carrier) | -0.003 | [-0.160, 0.155] | 0.971 | -0.006 |
| Household Income | -0.005 | [-0.020, 0.011] | 0.567 | -0.008 |
| Hypertension | 0.029 | [-0.005, 0.064] | 0.096 | 0.075 |
| Diabetes | 0.025 | [-0.029, 0.080] | 0.364 | 0.063 |
| Smoking | -0.023 | [-0.106, 0.060] | 0.589 | -0.056 |
| Non-English Speaking Background | 0.085 | [0.004, 0.167] | 0.04 | 0.221 |
| Exercise (none/mild vs moderate) | 0.014 | [-0.024, 0.052] | 0.463 | 0.037 |
| Exercise (vigorous vs moderate) | 0.048 | [-0.003, 0.098] | 0.066 | 0.122 |
| Lifetime Trauma:Gender (ref: Female) | -0.004 | [-0.042, 0.035] | 0.859 | -0.01 |
| Lifetime Trauma:Psychological Resilience | 0.014 | [-0.019, 0.046] | 0.41 | 0.033 |
| Gender (ref: Female):Psychological Resilience | 0 | [-0.036, 0.036] | 0.997 | 0 |
| Lifetime Trauma:Gender (ref: Female):Psychological Resilience | -0.002 | [-0.042, 0.037] | 0.916 | -0.004 |

Multiple R-squared: 0.4782; Adjusted R-squared: 0.4686; F-statistic: 49.49 on 20 and 1080 DF; p-value: < 2.2e-16

**References**

Anstey, K. J., Cherbuin, N., Christensen, H., Burns, R., Reglade-Meslin, C., Salim, A., Kumar, R., Jorm, A. F., & Sachdev, P. (2008). Follow-up of mild cognitive impairment and related disorders over four years in adults in their sixties: the PATH Through Life Study. *Dementia and Geriatric Cognitive Disorders*, *26*(3), 226-233. <https://doi.org/10.1159/000154646>

Eramudugolla, R., Mortby, M. E., Sachdev, P., Meslin, C., Kumar, R., & Anstey, K. J. (2017). Evaluation of a research diagnostic algorithm for DSM-5 neurocognitive disorders in a population-based cohort of older adults. *Alzheimer's Research & Therapy*, *9*(1). <https://doi.org/10.1186/s13195-017-0246-x>

Morris, J. C. (1993). The Clinical Dementia Rating (CDR). *Current version and scoring rules*, *43*(11), 2412-2412-a. <https://doi.org/10.1212/WNL.43.11.2412-a>

Petersen, R. C., Smith, G. E., Waring, S. C., Ivnik, R. J., Tangalos, E. G., & Kokmen, E. (1999). Mild cognitive impairment: clinical characterization and outcome. *Archives of Neurology*, *56*(3), 303-308. <https://doi.org/10.1001/archneur.56.3.303>

Sargent-Cox, K., Cherbuin, N., Sachdev, P., & Anstey, K. J. (2011). Subjective Health and Memory Predictors of Mild Cognitive Disorders and Cognitive Decline in Ageing: The Personality and Total Health (PATH) through Life Study. *Dementia and Geriatric Cognitive Disorders*, *31*(1), 45-52. <https://doi.org/10.1159/000322373>

Winblad, B., Palmer, K., Kivipelto, M., Jelic, V., Fratiglioni, L., Wahlund, L. O., Nordberg, A., Backman, L., Albert, M., Almkvist, O., Arai, H., Basun, H., Blennow, K., De Leon, M., Decarli, C., Erkinjuntti, T., Giacobini, E., Graff, C., Hardy, J.,…Petersen, R. C. (2004). Mild cognitive impairment - beyond controversies, towards a consensus: report of the International Working Group on Mild Cognitive Impairment. *Journal of Internal Medicine*, *256*(3), 240-246. <https://doi.org/10.1111/j.1365-2796.2004.01380.x>
